# Discovery of lipid-mediated protein–protein interactions in living cells using metabolic labeling with photoactivatable clickable probes†

**DOI:** 10.1039/d2sc06116c

**Published:** 2023-01-30

**Authors:** Roman O. Fedoryshchak, Andrii Gorelik, Mengjie Shen, Maria M. Shchepinova, Inmaculada Pérez-Dorado, Edward W. Tate

**Affiliations:** a Department of Chemistry, Molecular Sciences Research Hub, Imperial College London 80 Wood Lane London W12 0BZ UK e.tate@imperial.ac.uk; b The Francis Crick Institute 1 Midland Road London NW1 1AT UK

## Abstract

Protein–protein interactions (PPIs) are essential and pervasive regulatory elements in biology. Despite the development of a range of techniques to probe PPIs in living systems, there is a dearth of approaches to capture interactions driven by specific post-translational modifications (PTMs). Myristoylation is a lipid PTM added to more than 200 human proteins, where it may regulate membrane localization, stability or activity. Here we report the design and synthesis of a panel of novel photocrosslinkable and clickable myristic acid analog probes, and their characterization as efficient substrates for human *N*-myristoyltransferases NMT1 and NMT2, both biochemically and through X-ray crystallography. We demonstrate metabolic incorporation of probes to label NMT substrates in cell culture and *in situ* intracellular photoactivation to form a covalent crosslink between modified proteins and their interactors, capturing a snapshot of interactions in the presence of the lipid PTM. Proteomic analyses revealed both known and multiple novel interactors of a series of myristoylated proteins, including ferroptosis suppressor protein 1 (FSP1) and spliceosome-associated RNA helicase DDX46. The concept exemplified by these probes offers an efficient approach for exploring the PTM-specific interactome without the requirement for genetic modification, which may prove broadly applicable to other PTMs.

## Abstract 2

Metabolic labelling of a specific post-translational modification in cells with a bifunctional probe enables site-specific photocrosslinking, enrichment and identification of PTM-mediated protein–protein interactions through quantitative proteomics.

## Graphical abstract

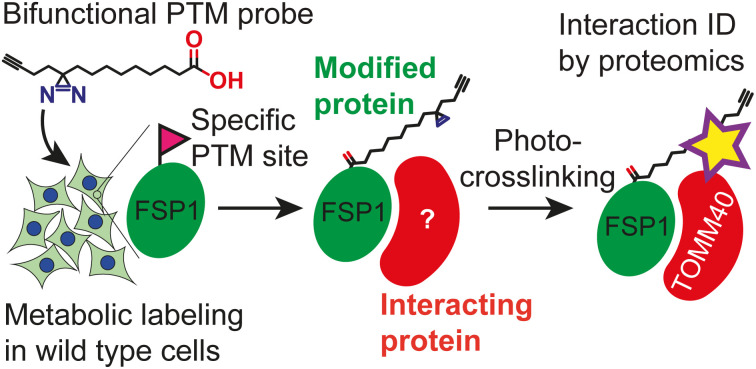

## Introduction

Protein–protein interactions (PPIs) are involved in virtually all aspects of cell physiology, from signaling and protein trafficking, to protein turnover and enzymatic activity. Post-translational modifications (PTMs) are chemical changes to protein structure that often regulate PPIs, but identifying PPIs mediated specifically by PTMs in intact cells is challenging due to the transient nature of PPIs and the fact that PTMs are regulated dynamically by transferases or hydrolases rather than being directly encoded in the amino acid sequence.^1^ Whilst proximity labeling proteomics has emerged as a powerful tool for analysis of protein adjacency in cells,^2^ direct identification of PTM-dependent PPIs remains challenging. In-cell photocrosslinking, most often exploiting a photolabile diazirine coupled with affinity enrichment and shotgun proteomics^3^ has been highly successful in identification of ligand–protein interactions^4–7^ and metabolite–protein interactions, including lipid–protein interactions.^8,9^ More recently, diazirine-containing amino acid mimics (so-called photo-leucine, photo-isoleucine, photo-methionine^10,11^ and, most notably, photo-lysine^12^) have been used to identify PPIs in cells *via* proteome-wide photocrosslinking^13,14^ or encoded through codon reassignment in a protein of interest.^15^ A synthetic clickable photocrosslinkable amino acid was recently used to profile PPIs specific to histone PTMs such as lysine acetylation and methylations in cell lysates.^16^ In one pioneering study, cells were metabolically labeled with a diazirine/alkyne dual-tagged palmitate probe, and photocrosslinking was employed to detect multimerization of *S*-palmitoylated IFITM3.^17^

**Fig. 1:**
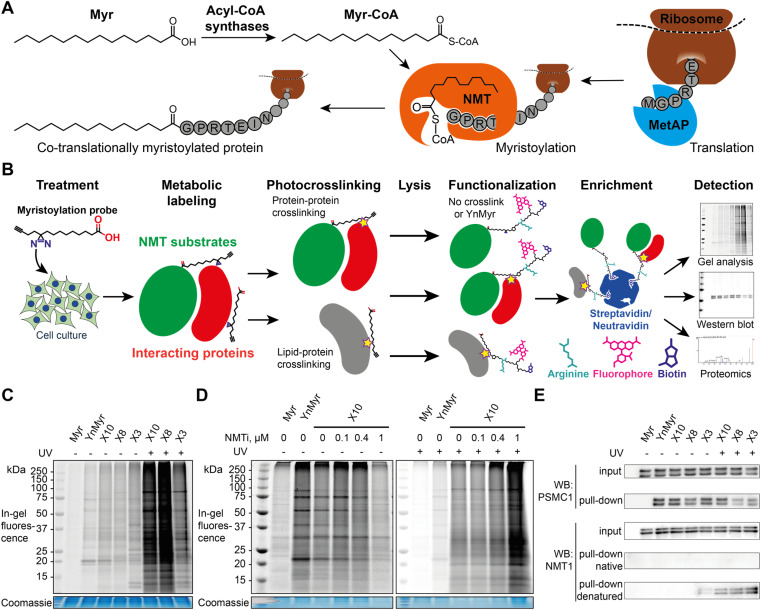
X3, X8 and X10 probes label myristoylated proteins in cells. (A) Diagram showing cellular transformations leading to protein myristoylation. Initiator methionine aminopeptidase (MetAP) (co-translational myristoylation, shown) or an endoprotease (post-translational myristoylation) reveal an N-terminal glycine *via* proteolysis, which is then myristoylated by NMT. (B) Myristoylated proteome labeling strategy: diazirine probe is incubated with cells in culture and incorporated into the proteome, photocrosslinked by irradiation at 365 nm, and following lysis alkyne groups are functionalized with dye and/or biotin labels, optionally including a trypsin-digestible arginine linker. Proteins can be visualized by in-gel fluorescence or enriched on streptavidin/neutravidin beads for western blotting and proteomics. (C) Cells were treated with a panel of myristate analogs and visualized by in-gel fluorescence. A myristoylated protein labeling pattern is observed for all probes without UV-irradiation (UV), YnMyr (5 μM) and X3/X8/X10 (100 μM each), whilst X3/X8/X10 probes are crosslinked to cellular proteins upon UV irradiation. (D) IMP-366 (NMTi) treatment inhibits myristoylated proteome labeling by X10; upon UV-irradiation, X10 labeling is NMT-independent and is likely dominated by lipid–protein crosslinks. For the unadjusted contrast range see Fig. S1.† (E) Cells were treated with a panel of myristate analogs, and labeled proteins enriched on streptavidin-conjugated beads. Western blots of NMT substrate PSMC1 before (input) and after enrichment (pull-down) indicate labeling with X3/X8/X10 (100 μM each) probes comparable to YnMyr (5 μM). Equivalent blotting for NMT1 labeling (a buried site) shows photocrosslinking-dependent enrichment only with denaturation at 95 °C pre-CuAAC ligation.

*N*-myristoylation is a lipid PTM in which the 14-carbon saturated fatty acid myristate (Myr, C14:0) is transferred from myristoyl-coenzyme A (Myr-CoA) to the N-terminal glycine of substrate proteins in cells, catalyzed by *N*-myristoyl transferases (NMT1 and NMT2 in humans). Myr-CoA is bound in a well-defined hydrophobic pocket, triggering binding of up to 10 residues of the N-terminus of a substrate protein in the substrate-binding groove.^18,19^ Myristate (Myr) is transferred to the substrate protein N-terminus followed by the release of product myristoylated protein and CoA (Fig. 1A), in a catalytic cycle recently described at high resolution.^20^ Myristoylation occurs both co-translationally and post-translationally in cells; it promotes dynamic association with cellular membranes which can be stabilized by additional signals such as further lipidation (often *S*-acylation), basic amino acids adjacent to the N-terminus or protein–protein interactions (PPIs), and reversed through further PTMs, soluble chaperone binding or an intramolecular change in conformation in a so-called myristoyl switch.^19,21^ There are only a few known examples of PPIs mediated directly by myristoylated N-termini; for example, myristate-mediated interaction of CHP1 to activate acyltransferase GPAT4,^22^ the well-defined myristate-binding pocket in chaperones UNC119A and UNC119B which recognize specific myristoylated clients for intracellular trafficking,^23,24^ and heme oxygenase 2, which can bind both free myristic acid and the myristoylated HIV-1 protein Gag.^25^

Through structure guided design and optimization of novel myristate-based probes with dual diazirine and alkyne functionality, we report a technology platform to profile *N*-myristoylation-mediated PPIs in wild-type cells at the whole proteome level, without genetic modification. Coupling these probes with enrichment and proteomic mass spectrometry, we identify multiple novel interactors for a series of myristoylated proteins, including ferroptosis suppressor protein FSP1 and spliceosome-associated RNA helicase DDX46. These data validate a novel tool for profiling myristoylation-mediated PPIs and exemplify a potentially generalizable approach for discovery of interactomes dependent on specific PTMs.

## Results

### Design and synthesis of photocrosslinkable clickable myristate probes

Previously described photoactivatable myristate analogs are not suitable for metabolic incorporation into the myristoylated proteome in human cells due to the bulky crosslinking moiety rendering them incompatible as human NMT substrates.^26,27^ We elected to adopt a diazirine as a photoactivatable moiety, since this group is only slightly more sterically demanding than a methylene group and more likely to be accepted by the *N*-myristoylation machinery. Upon UV irradiation, the diazirine group may eliminate nitrogen to generate a reactive carbene which readily crosslinks to neighboring molecules,^28,29^ and can be used for *de novo* mass-spectrometry identification of crosslinked proteins in combination with a bioorthogonal tag to enable click chemistry ligation and affinity purification.

**Scheme 1:**
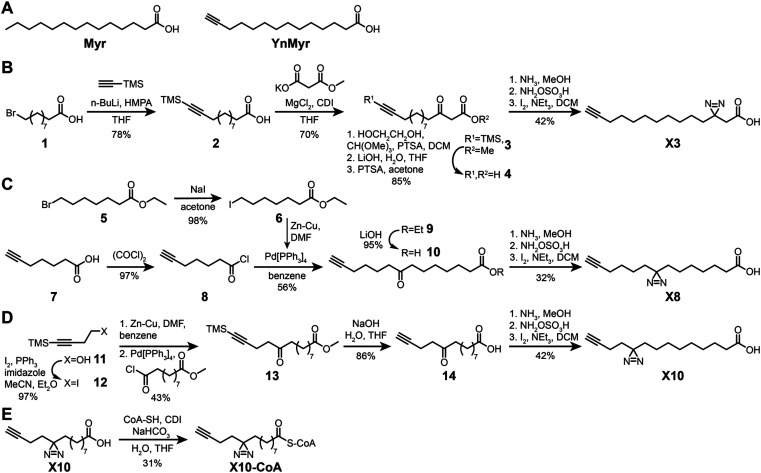
Synthesis of photocrosslinkable clickable myristate probes. (A) Left, myristic acid (Myr). Right, YnMyr probe. (B–E) Synthesis schemes for X3 (B), X8 (C), X10 (D) and X10-CoA (E). Full synthesis and characterization details are provided in the ESI.†

We designed three photocrosslinkable clickable analogs of myristic acid, X3, X8 and X10, based on the structure of the alkyne-functionalized probe YnMyr which mimics myristic acid,^30^ and has previously been applied to the identification of the *N*-myristoylated proteome and functional studies of NMT inhibition^31,32^ (Myr, Scheme 1A). The probes contain the diazirine moiety at specific positions along the 14-carbon fatty acid chain which we predicted might accommodate this group based on inspection of our previously reported structure of the human NMT1 (HsNMT1):Myr-CoA complex (PDB:4C2Y). Each of the analogs also carries an ω-alkyne group between carbons 13 and 14, similar to YnMyr, to enable functionalization *via* copper(i) catalyzed azide–alkyne cycloaddition (CuAAC) ligation (click chemistry) with various azide-bearing reagents.^31–33^ Syntheses of probes X3, X8 and X10 were designed such that the transformation of ketone to diazirine was undertaken at the end of the synthesis (Schemes 1B–D). For X3 synthesis, 10-bromodecanoic acid was reacted with trimethylsilylacetylene to yield the trimethylsilyl-protected alkyne analogue of lauric acid 2,^34^ followed by condensation and homologation using the Masamune procedure^35^ to obtain the ketone precursor for X3 (Scheme 1B). In contrast, the synthesis of ketone precursors 10 and 13 for probes X8 (Scheme 1C) and X10 (Scheme 1D) respectively required a more challenging sp^2^–sp^3^ C–C coupling; Pd(0) catalyzed coupling was performed by adding the respective chloroanhydrides to alkyl zinc iodides generated *in situ* from aliphatic alkyl iodides by Cu–Zn couple,^36^ resulting in desired ketone precursors of X8 and X10 in moderate yields. Each ketone precursor was treated with ammonia followed by hydroxylamine-*O*-sulfonic acid to obtain the corresponding diaziridine, which was oxidized to the diazirine using iodine in 20–42% yields, similar to previously described protocols.^37^

### X3, X8, and X10 probes metabolically label proteins in an NMT-dependent manner

The alkyne-bearing probe YnMyr is readily incorporated into the myristoylated proteome by incubation with cultured cells and, following lysis, biotin- and/or fluorophore-functionalized capture reagents can be ligated to YnMyr-tagged proteins to enable subsequent in-gel visualization and/or affinity enrichment and proteomic analysis.^31,38,39^ A similar workflow was used in this study with the additional step of in-cell photocrosslinking for X3/X8/X10 probes (Fig. 1B).

To test the ability of diazirine containing probes to incorporate into the myristoylated proteome, each photocrosslinkable probe X3/X8/X10 at 100 μM, or YnMyr at 5 μM concentration (yielding equal labeling intensity), or untagged control myristic acid Myr, were added to HeLa cells in culture and incubated for 24 h to allow incorporation. This treatment was followed by *in situ* irradiation with UV light at 365 nm (+UV; 5 min) to initiate in-cell diazirine photoactivation, or without irradiation (−UV) in control samples. CuAAC ligation of alkyne-tagged proteins to azido-TAMRA (AzT)^33^ was used for in-gel fluorescence visualization (Fig. 1C). Without UV-irradiation (−UV), protein labeling for X3/X8/X10 resembled YnMyr, suggesting similar proteins are labeled independently of the presence of the diazirine group. Interestingly, X3 showed some additional bands even without UV irradiation which we hypothesize may be due to higher reactivity or instability of the β-diazirine, differential metabolic activation, or the position of the diazirine being relatively less buried when the probe is located in the membrane which may lead X3 to label other proteins compared to X8/X10. Upon UV-irradiation (+UV), the number and intensity of fluorescent bands was greatly increased, attributed to lipid–protein crosslinking derived from non-covalently bound X3/X8/X10 fatty acids which greatly increase the range of proteins visualized in-gel (Fig. 1C and D, and S1†), potentially further augmented by metabolism of X3/X8/X10 into *e.g.* chain-extended fatty acids or other more complex lipid species. To confirm the dependence of labeling on NMT activity, we examined the impact of a potent and selective NMT inhibitor (NMTi) IMP-366 (ref. 40 and 41) on labeling by a representative probe (X10); with increasing NMTi concentration the majority of fluorescently labeled bands disappeared, indicating a decrease in NMT-dependent metabolic labeling of myristoylated proteins, similarly to previously published data.^31,38^ Lack of a clear NMTi-mediated decrease in labeling *via* myristoylated proteins under UV irradiation was expected due to the presence of the far more intense signal from lipid–protein crosslinking (Fig. 1D and S1†). To confirm labeling of a specific NMT substrate, we ligated proteins tagged with X3, X8 or X10 to an azido-TAMRA-biotin (AzTB) capture reagent followed by streptavidin-conjugated magnetic bead enrichment to evaluate metabolic labeling and capture of PSMC1, a previously identified myristoylated protein and a component of the proteasome complex.^31^ Enrichment of PSMC1 was observed with all alkynylated probes with or without UV irradiation, but no enrichment in the absence of alkyne in the myristic acid control experiment was observed (Fig. 1E); however, higher molecular weight bands on the blot signifying protein–protein crosslinking in the UV-irradiated samples were not observed for PSMC1 (data not shown).

### X3/X8/X10 probes form stable crosslinks with NMT in cells upon UV irradiation

As a substrate in the myristoylation reaction, Myr-CoA interacts with NMT non-covalently, and therefore NMT1 and NMT2 are not labeled in experiments with YnMyr enrichment. However, irradiation of X3/X8/X10-treated samples induced crosslinks between NMT1 and metabolically modified X3/X8/X10-CoA probes, which could be detected by western blotting (Fig. 1E). Consistent with our expectations, without denaturation of proteins in the cell lysate prior to CuAAC, the alkyne functionality appears to be buried inside NMT1 and is inaccessible for ligation to azido-biotin. Upon protein denaturation by heating (95 °C) the lysates in the presence of 1% sodium dodecyl sulfate (SDS), it was possible to ligate and enrich NMT1 photo-crosslinked to each probe; again, probe X3 showed evidence of background crosslinking even without UV irradiation, supporting the hypothesis that this probe has greater inherent reactivity than X8 or X10.

### X3/X8/X10 probes label NMT substrates proteome-wide

**Fig. 2:**
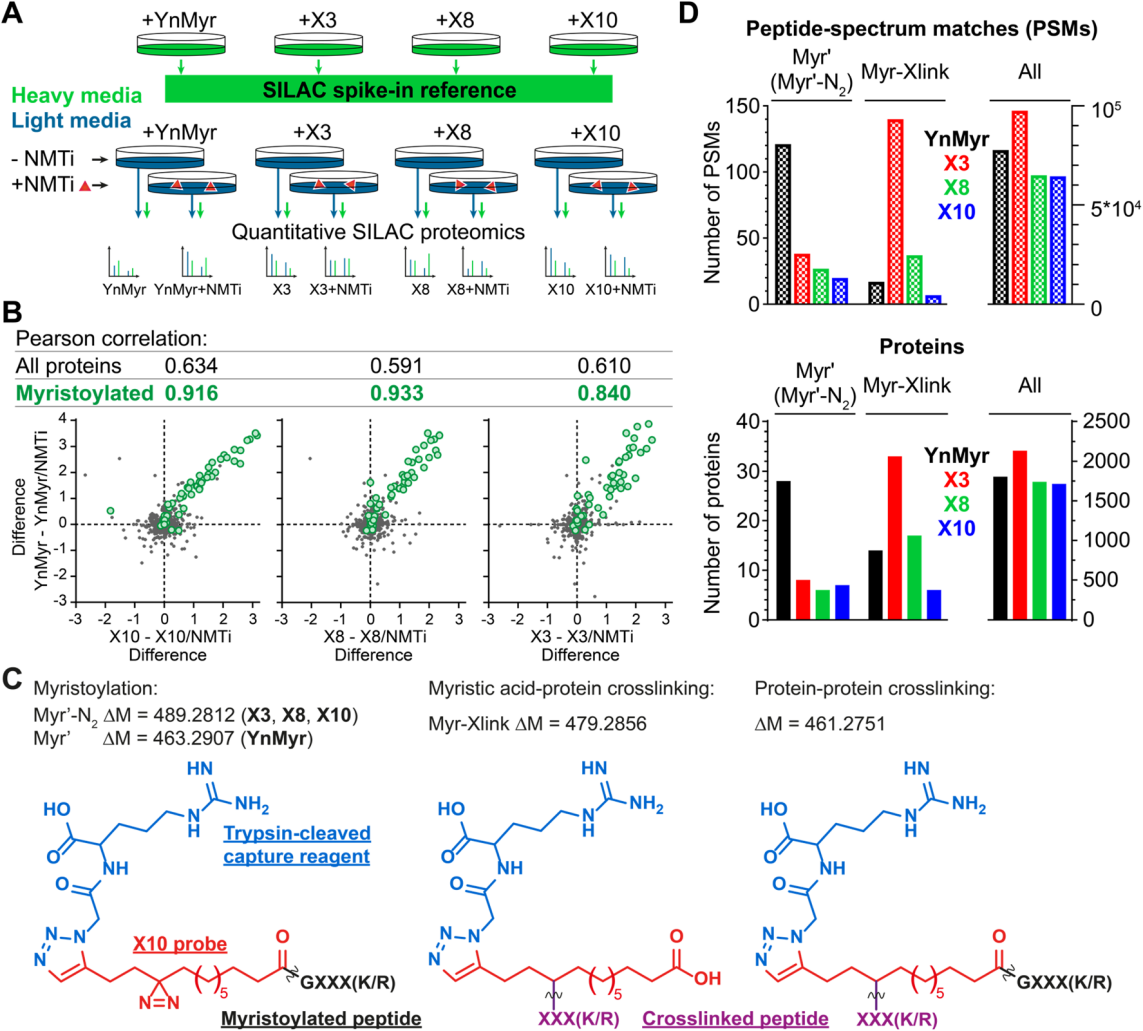
Incorporation of photocrosslinkable clickable probes into the myristoylated proteome. (A) Experimental design for quantitative SILAC proteomics validating myristoylated proteome labeling by X3/X8/X10 (100 μM each) compared to positive control YnMyr (5 μM) and negative controls obtained by 0.5 μM NMTi treatment. Treated cells were lysed, ligated to an azido-arginine-biotin capture reagent and enriched on neutravidin-agarose resin. (B) Pearson correlation between myristoylated protein enrichment (green) using either photocrosslinkable clickable probes X3/X8/X10 or YnMyr. Grey – background (non-myristoylated) proteins. Axes are differences in protein enrichment between vehicle (DMSO) and NMTi-treated samples for X3/X8/X10 (*X*-axes) and YnMyr (*Y*-axis). (C) Structures and masses of PTMs searched to identify X3/X8/X10/YnMyr-related modifications. (D) Left – Total number of Myr′, Myr′-N2, and Myr-Xlink (see (C)) modified peptide-spectrum matches (PSMs, top) or proteins (bottom) identified using PEAKS search engine^42^ for X3/X8/X10 or YnMyr. Right – All PSMs or proteins detected in the experiment for a given probe.

Using YnMyr as a reference point for the myristoylated proteome,^31^ we assessed proteome-wide labeling with each of the X3/X8/X10 probes using neutravidin enrichment and quantitative SILAC proteomics. An equal mixture of lysates from four HeLa cell cultures treated with each probe (YnMyr, X3, X8 and X10) grown in heavy arginine and lysine (R10K8)-containing media was used as spike-in standard to normalize quantification across experiments. Cultures grown in light media (R0K0) were treated with each myristate analog and with either 0.5 μM NMT inhibitor IMP-366 (ref. 40) or DMSO vehicle, and heavy lysate spike-in added to each lysate to enable quantitative analysis of proteome-wide NMT-dependent probe incorporation (Fig. 2A, ESI Table 1†). Ligation to an azido-arginine-biotin capture reagent and enrichment for probe-tagged proteins on neutravidin-agarose beads demonstrated that the labeling of myristoylated proteins with X3/X8/X10 or YnMyr decreased on NMTi treatment. Quantitative comparisons with/without NMTi show a Pearson correlation >0.9 between the samples treated with YnMyr*vs.*X10 or X8, and 0.84 *vs.*X3 (Fig. 2B), consistent with diazirine derivatives labeling myristoylated proteins equivalently to YnMyr in cells.

We used the PEAKS *de novo* sequencing analysis package^42^ to identify probe-modified peptides in MS/MS spectra, denoting Myr′ for YnMyr-labelled or Myr′-N2 for X3/X8/X10-labelled N-terminal peptides, respectively; and Myr-Xlink for X3/X8/X10 probe-to-protein crosslinks (Fig. 2C). We identified 121 peptide spectrum matches (PSMs) with Myr′ modification corresponding to 28 known myristoylated proteins. Probes X3, X8 and X10 were less efficient in direct detection of lipidation, although Myr′-N2 modifications for each diazirine-containing probe were detected on 6–8 proteins, with multiple PSMs per identification (Fig. 2D).

### Identification of X3/X8/X10-direct-to-protein crosslinks

Intriguingly, several non-myristoylated proteins were enriched in the X3/X8/X10 samples compared to YnMyr (Fig. S2A–C†). X8 and X10 probes consistently enriched for components of the fatty acid beta-oxidation pathway, such as long-chain-fatty-acid-CoA ligases (ACSL3, ACSL4), acyl-CoA dehydrogenases (ACAD9, ACADM), enoyl-CoA hydratase (ECHS1), 3-hydroxyacyl-CoA dehydrogenase (HSD17B4) and 3-ketoacyl-CoA thiolases (ACAA1, ACAA2). These proteins are involved in fatty acid and acyl-CoA metabolism, and apparent enrichment may result in part from changes in gene expression in response to treatment with a relatively high concentration of fatty acid probe (100 μM X8/X10*vs.* 5 μM YnMyr), or from metabolic activation in the absence of UV light.

**Fig. 3:**
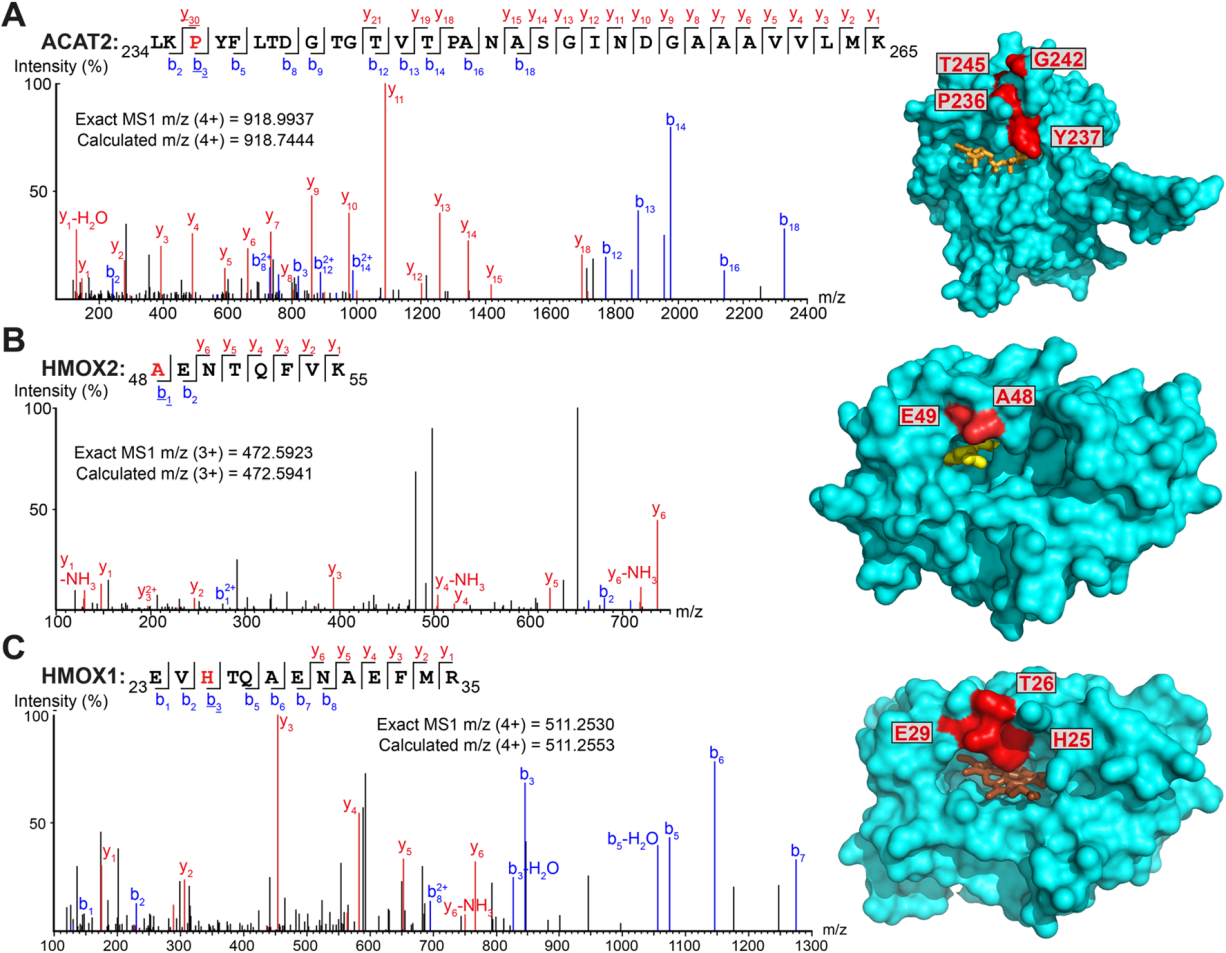
Myristate probes X3/X8/X10 form crosslinks with myristate-binding proteins. (A–C) Representative MS/MS fragmentation spectra and structural positions of crosslinked amino acids (red) for ACAT2 ((A), PDB:1WL4, orange – acetyl-CoA), HMOX2 ((B), PDB:5UC9, yellow – myristic acid) and HMOX1 ((C), PDB:1N45, brown – heme) structures.

Numerous additional proteins were enriched in X3-labeled samples, and we considered the possibility that some of these proteins may be crosslinked to X3 non-specifically (without UV irradiation). We searched for PSMs corresponding to MyrXlink peptides (Fig. 2C) on all residues and found 140 PSMs for 33 proteins with Myr-Xlink modification in addition to 35 PSMs for 8 proteins corresponding to Myr′-N2 modification (Fig. 2D). Due to expected inaccuracies in the analysis of large datasets, the peptide-spectrum matching algorithm erroneously identified 16 Myr Xlink PSMs on 14 proteins in the YnMyr-treated samples, which we used to establish the rate of false-positive identifications. Similarly low numbers of Myr′-Xlink PSMs were observed in non-irradiated X8- and X10-treated samples. Taken together, these data indicate a degree of baseline reactivity for the diazirine group in β-position to the carbonyl consistent with the crosslinking ‘leakage’ of X3 observed under non-irradiated conditions by western blotting (Fig. 1E), whereas X8- and X10 probe diazirine groups appear to be unreactive without UV-irradiation. Cytosolic acetyl-CoA acetyltransferase (ACAT2), a protein involved in lipid metabolism, is among the proteins modified by X3 and quantitatively enriched compared to YnMyr (Fig. S2A†). We found 14 PSMs linking X3 to ACAT2 with modifications on four residues (P236, Y237, G242, T245) located close to the CoA binding site (Fig. 3A and S3A,† PDB:1WL4 (ref. 43)). Similarly, heme oxygenase 2 (HMOX2), also known as a myristate-binding protein,^25^ crosslinked with X3 at two distinct sites (A48 and G49, 10 Myr-Xlink PSMs) which map to the myristate binding site in the published myristic acid-HMOX2 crystal structure (Fig. 3B and S3B,† PDB:5UC9 (ref. 25)). Heme oxygenase 1 (HMOX1) is structurally homologous to HMOX2, and we identified 13 Myr-Xlink PSMs for X3 on residues (H25, T26, E29) adjacent to the HMOX1 heme-binding site (Fig. 3C and S3C,† PDB:1N45 (ref. 44)). This novel HMOX1 myristate binding site is found at the canonical heme site, closely analogous to HMOX2 despite HMOX1 and HMOX2 sequence divergence. We have thus precisely identified several examples of the unmetabolized X3 probe specifically crosslinked to proteins possessing myristate binding sites. Although not the main focus of this study, these data highlight the potential of diazirine-based probes for mapping known and novel protein/lipid or protein/acyl-CoA binding sites in a manner consistent with a previous study of histone PTMs.^16^

### Myristoylated proteins labeled with X3 and X10 probes form photocrosslinks with interactors

To explore whether probe crosslinking could capture PPIs specific to *N*-myristoylated proteins in addition to binary protein–lipid interactions, we analyzed the impact of NMTi on crosslinking to identify non-myristoylated proteins captured by virtue of NMT-mediated probe incorporation (ESI Table 2†), focusing on UV-irradiated X3- or X10-treated cells with or without NMTi. SILAC proteomic analysis identified PSMs that recapitulated crosslinking to ACAT2 (residues P236, G242 and G244) and HMOX2 (A48, G49) in X3 samples, among other proteins, although PSMs for peptides crosslinked to X10 were not identified, highlighting differences between these probes (see the Discussion). As before, known myristoylated proteins were identified in both X3- (Fig. S3D†) and X10- (Fig. S3E†) treated samples, with their enrichment significantly reduced upon NMTi treatment. In addition, we observed a set of non-myristoylated proteins (Fig. S3F†) enriched specifically in non-NMTi samples, representing candidates for myristoylation-dependent PPIs. The number of significantly co-enriched proteins was greatly increased in UV-treated samples in comparison to the previous analysis without UV-irradiation (Fig. S3G and H†). Identification of peptide-to-peptide crosslinks by MS/MS proved challenging, however, and we were unable to confidently resolve direct interactions between myristoylated proteins and enriched non-myristoylated proteins at the whole proteome level. Therefore, we turned to a targeted approach to identify PTM-dependent interactions of specific myristoylated proteins.

### X10-CoA is an efficient human NMT substrate *in vitro* and is accommodated in the Myr-CoA pocket

**Fig. 4:**
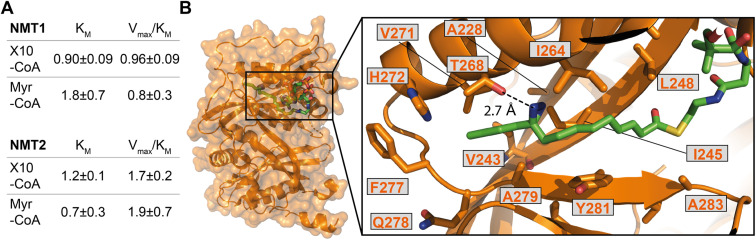
X10-CoA is an NMT1/2 substrate. (A) Rates of enzymatic transfer of Myr or X10 by HsNMT1 or HsNMT2 to a synthetic peptide measured *in vitro* by CPM assay;^45^ both natural and artificial substrates show similar catalytic efficiencies (*V*_max_/*K*_M_ ± SD, RFU min^−1^ μM^−1^ × 10^6^) and Michaelis constants (*K*_M_ ± SD, μM); (B) crystal structure of HsNMT1 (orange) in complex with X10-CoA (green) showing the overall structure and an enlarged view of the X10 moiety (chain A) with a potential hydrogen bond between the diazirine and T268. Color code: blue (nitrogen), red (oxygen), yellow (sulfur), and orange (protein structure).

We next sought to confirm X10 as an optimal photoactivatable NMT substrate biochemically, and structurally, by exploring the binding mode of its activated CoA thioester form (X10-CoA) by X-ray crystallography. Thioester X10-CoA (Scheme 1E) was generated by conjugation of X10 and coenzyme A thiol (CoA-SH) in the presence of 1,1′-carbonyldiimidazole (CDI) and base.^30^ Enzyme kinetics were analyzed using an *in vitro* assay which detects CoA-SH release through a fluorogenic reaction with 7-diethylamino-3-(4-maleimidophenyl)-4-methylcoumarin (CPM),^45^ comparing the activity of HsNMT1 and HsNMT2 in transferring the fatty acid moiety of X10-CoA*vs.* the native substrate Myr-CoA to a model peptide based on a c-Src N-terminal peptide (16 μM H-GSNKSKPK-NH_2_). The catalytic efficiency of X10-CoA and Myr-CoA was very similar for both enzymes, NMT1 and NMT2, with a small change in *K*_M_ (Fig. 4A and S4A†), indicating excellent biochemical compatibility between X10 and human NMT1/2 *in vitro*.

To gain insights into the binding mode of X10-CoA in the active site of NMT, we obtained crystals of HsNMT1 in complex with X10-CoA which enabled us to solve the structure at 2.37 Å resolution (Fig. 4B and S4B–D, and ESI Table 3†). The two HsNMT1:X10-CoA complexes per asymmetric unit exhibited the same fold (root mean-square deviation (RMSD) of 0.497 Å for 354 Cα atoms), whilst structural superimposition of this complex with our previously reported HsNMT1:Myr-CoA structure (PDB:4C2Y^31^) confirms an equivalent HsNMT1 fold in complexes with X10-CoA or the natural substrate (RMSD 0.587 Å over 355 Cα atoms, Fig. S4B†). Both X10-CoA molecules were well-defined in the electron density, which enabled us to model X10-CoA, including the diazirine group, unambiguously. Consistent with the capacity of X10-CoA to mimic the natural substrate, X10-CoA binds at the Myr-CoA binding site with the X10 moiety lying along the myristate-binding groove in a very similar conformation to the Myr-CoA myristate (Fig. S4C†). The myristate binding site is highly hydrophobic except for the presence of T268 and H272 residues, and interestingly the diazirine is situated at hydrogen bonding distance from the T268 side-chain hydroxyl, supporting selection of X10 as an optimal substrate (Fig. 4B).

### X10 probe enables *de novo* identification of PTM-dependent protein–protein interactions in live cells

**Fig. 5:**
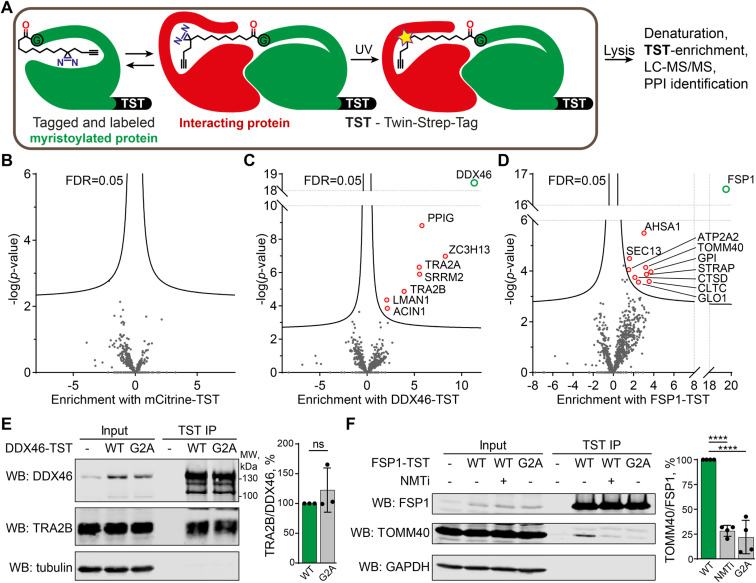
Identification of PPIs by photocrosslinking. (A) Scheme of X10 photocrosslinking experiment to identify PPIs of TST-tagged myristoylated proteins. (B–D) Fold protein enrichment from cells transfected with mCitrine-TST (B), DDX46-TST (C) or FSP1-TST (D) in triplicates, relative to all other samples combined (19 proteins × 3 biological replicates) on the *X*-axis, plotted against statistical significance (*Y*-axis). (E and F) Validation of DDX46 (E) and FSP1 (F) interactors. Pulldowns for DDX46-TST and FSP1-TST were probed with TRA2B and TOMM40 antibodies, respectively. α-Tubulin or GAPDH were used as loading controls. Normalization was performed by dividing the TRA2B or TOMM40 antibody signal by the DDX46 or FSP1 signal, respectively. Data are shown as mean ± s.e.m., *n* = 3–4. ****p = 0.0001, ns – no significant difference, calculated by the Student's *t*-test (two-tailed, unpaired).

We chose to focus on X10 for targeted identification of myristoylation-dependent PPIs, as among the three probes it shows the highest efficiency and fidelity for myristoylation, with optimal NMT substrate properties and a well-defined binding mode (see above). HEK293 cells were transfected with twenty different myristoylated proteins engineered to bear a C-terminal Twin-Strep-tag (TST) for affinity purification, or mCitrine-TST as a non-myristoylated negative control (for the full list of protein constructs used, and related proteomics data, see ESI Table 4†). Upon transfection, cells were treated with 100 μM X10 for 24 h before UV-irradiation. Cells were lysed with a buffer containing 1% SDS and heated at 95 °C for 10 min to eliminate non-covalent PPIs, after which myristoylated TST-tagged proteins were enriched at ambient temperature with Strep-Tactin resin, digested with trypsin and analyzed by proteomics (Fig. 5A). Label-free quantification was used to determine the fold change of proteins enriched by a given myristoylated protein construct against the combined data of the 19 other protein constructs in the overall experiment. As expected, the corresponding TST-tagged constructs were the most strongly enriched proteins in each case, and no proteins were significantly enriched in the mCitrine control, demonstrating the specificity of TST enrichment and the dependence of photocrosslinking on the incorporation of X10 (Fig. 5B). However, several myristoylated protein constructs enriched putative photocrosslinked interactors (ESI Table 4), with DDX46 (Fig. 5C) and FSP1 (Fig. 5D) showing the highest hit rates.

Myristoylated ATP-dependent RNA helicase DDX46 (homologue of *Saccharomyces cerevisiae* PRP5) is localized in the nucleus and has an essential role in splicing, where it participates in the formation of the 17S U2 snRNP complex, a subunit of the spliceosome A and E complexes.^46^ Consistent with DDX46 localization and function, proteins ACIN1, PPIG, SRRM2, TRA2A/B, and ZC3H13 identified in our data as DDX46 interactors are all components of RNA splicing and have high confidence as interactors based on previously reported interactome data^47^ (Fig. S5†). We further analyzed the interaction of DDX46 with TRA2B, an RNA-binding protein involved in control of pre-mRNA splicing.^48,49^ DDX46-TST or its myristoylation-deficient G2A mutant was transfected and immunoprecipitated from HEK293 cells on Strep-Tactin resin, showing that both the wild type (WT) and G2A mutant interacted with TRA2B to a similar extent (Fig. 5E). These data indicate that the DDX46-TRA2B interaction is mediated by structural and sequence determinants other than myristoylation, but nevertheless demonstrate the utility of X10 as an enzymatically incorporated photoaffinity probe for identification of novel interactors in intact cells.

FSP1 (ferroptosis suppressor protein 1) is a myristoylated oxidoreductase that converts coenzyme Q_10_ into its reduced form at the membrane, preventing lipid oxidation by quenching reactive oxygen species, and suppressing ferroptosis, a type of iron-dependent cell death.^50,51^ We identified several FSP1-interacting proteins associated with mitochondria and oxidative stress, including peroxisomal oxidase GLO1, oxidative stress-associated ATPase ATP2A2 and protease cathepsin D among others. We used a similar co-immunoprecipitation approach to validate the interaction of FSP1-TST with TOMM40, which is a mitochondrial protein that facilitates protein import into mitochondria,^52,53^ and is itself also myristoylated. TOMM40 co-immunoprecipitated with FSP1, which was strongly suppressed in a myristoylation-deficient FSP1 G2A mutant (to 22% of WT FSP1), whilst NMT inhibition in the presence of wild-type FSP1 similarly reduced immunoprecipitation to 28% of WT (Fig. 5F). These data support a novel interaction between two mitochondrial myristoylated proteins FSP1 and TOMM40, for which FSP1 myristoylation is essential.

## Discussion

Myristoylation is an essential cellular process, inhibition of which has been demonstrated as a potential treatment in a variety of infectious diseases^38,54^ and cancer.^55,56^ Besides providing target proteins with lipophilicity, myristoylation can also lead to changes in protein conformation and thus affect PPIs. However, only a few myristate-mediated PPIs have been described. We sought to identify weak and transient myristate-mediated PPIs with minimal perturbation in intact cells using *in situ* photocrosslinking through diazirine- and alkyne-modified myristate probes X3, X8 and X10.

Probes X3/X8/X10 are diazirine-modified derivatives of the clickable myristate analog YnMyr, which has been applied extensively in cells to characterize the myristoylated proteome and study the effects of NMT inhibition. Our data show that these probes mimic myristate in cells through metabolic incorporation into NMT substrates, a feature we attribute to the small size and minimal perturbation caused by diazirine and alkyne modification. Like YnMyr, these probes must first be activated as coenzyme A derivatives, likely through long-chain-fatty-acid-CoA ligases 3 and 4 (ACSL3/4) that we found crosslinked with X3/X8/X10 (Fig. S2A–C†). 100 μM X3/X8/X10 was required to reach the same level of myristoylated protein labeling achieved with 5 μM YnMyr, which we hypothesize is either due to more efficient catabolism of diazirine-containing probes or to a lower efficiency of uptake or activation by acyl-CoA ligases. X3/X8/X10 probes are nevertheless recognized as substrates by native NMTs in cells, while in enzyme activity assays X10-CoA shows equal catalytic efficiency with HsNMT1 and HsNMT2 to the native substrate Myr and binds in the Myr-CoA binding site of HsNMT1 as determined by X-ray crystallography. Furthermore, we find that myristoylated proteins labelled with probes in cells can participate in PPIs and form crosslinks to respective interactors.

Myristoylation presents a convenient PTM system to apply photocrosslinking proteomics approaches to selected proteins, offering some advantages complementary to alternative approaches. Myristoylated proteins are typically stoichiometrically labeled at the point of synthesis, with no mechanism for subsequent removal, in contrast to reversible *S*-acylation, whilst photo-amino acid labeling requires either laborious and disruptive introduction of amber codon suppression or indiscriminate whole-proteome photo-amino acid labeling by photo-substitutes of natural amino acids. On the other hand, metabolic incorporation of X3/X8/X10 into the myristoylated proteome may result in labeling of >100 natively myristoylated human proteins, and the resulting photocrosslinks may be due to interaction of one or several myristoylated proteins with an interactor. In future, it may prove possible to deconvolute such interactions at the whole proteome level through identification of photocrosslinked peptide sequences from MS/MS spectra. Identification of such crosslinks proteome-wide is currently only possible using specialized chemical crosslinking reagents,^57^ and would likely require advances in mass spectrometry hardware (quality of spectra) and software (searching multi-dimensional MS/MS space) to be used with our current reagents. Another complication of using metabolic labeling to study *N*-myristoylation with myristic acid analogs is their propensity to metabolize and be incorporated into other lipid species. However, we took advantage of this property of X3/X8/X10 probes to identify numerous lipid–protein interactions proteome-wide, in line with previous research,^58^ alongside precise sites of fatty acid–protein binding through crosslinking analysis by *de novo* sequencing;^59^ in contrast, standard database searches (*e.g.* in MaxQuant^60^) failed to identify crosslinks using the same search parameters.

When analyzing fatty acid–protein crosslinks, we noticed an increased baseline reactivity of the diazirine group in β-position to the carboxyl group (Fig. 1E, 2D and S2A†). To our knowledge, such a juxtaposition of functional groups has been reported only once previously, and was not applied for protein photocrosslinking,^61^ with the majority of recent reports placing a diazirine group γ- to a carboxyl/amide group, including the so-called “minimalist photocrosslinkers”,^62^ or in aromatic trifluoromethyl diazirines. Our observation that β-diazirine carboxylic acid X3 undergoes spontaneous crosslinking may be explained by diazirine conversion to diazoalkane by protonation,^63^ providing a mechanism by which X3 is more susceptible to non-irradiative decomposition: the weakly acidic proton of the neighboring carboxyl is ideally placed to form a hydrogen bond with a diazirine lone pair and thus facilitates transition to a reactive *N*-protonated carbocationic diazolakane species. Diazoalkanes may also be converted to reactive carbenes upon 365 nm UV-irradiation. Although the identification of many directly modified binding sites demonstrates that the probes can be incorporated without additional metabolism, our data do not exclude the possibility that variations in labeling efficiency between probes may result from the varying position of the diazirine relative to the membrane between X3/X8/X10, or differences in metabolic activation between the probes.

Comparing the X3/X8/X10 probes, we found that X10 offers superior and consistent myristoylated proteome labeling on par with YnMyr. Using X10, we were able to capture and identify myristate PTM-mediated interactions by transfection and affinity-tag enrichment of a protein of interest, whereby the diazirine functionality of X10 was used for crosslinking but the alkyne was left unreacted. Interactions were identified and validated for myristoylated FSP1 and DDX46 in addition to known interactions, for example, for myristoylated Src kinase with KHDRBS1 (ref. 64) and HNRNPK^65^ (ESI Table 4†). Whilst the majority of DDX46 interactors identified in the present study are RNA splicing-associated proteins in line with its role as a component of the U2 snRNP complex, our data suggest a potential wider role for DDX46 in RNA splicing. For example, we confirmed an interaction of DDX46 with pre-mRNA-binding protein TRA2B which regulates alternative splicing, and these findings may inform investigation of functional roles for DDX46 interaction with TRA2B as well as TRA2A, ACIN1, PPIG, SRRM2 and ZC3H13 in future studies of splicing regulation. FSP1 was recently identified as a novel ferroptosis suppressor protein and potential target for cancer therapy;^50,51^ however, understanding of the FSP1 interaction network is currently limited. Here, we have identified nine probable FSP1-interacting proteins, and confirmed a physical interaction with TOMM40. FSP1 myristoylation appears necessary for the FSP1–TOMM40 interaction, although it remains to be established whether the interaction is direct or through co-localization. Further investigation will be required to dissect the role of FSP1–TOMM40 interaction in normal cell state and ferroptosis.

## Data availability

RCSB PDB accession for HsNMT1:X10-CoA crystal structure: 5NPQ. The mass spectrometry proteomics data have been deposited to the ProteomeXchange Consortium *via* the PRIDE partner repository with the dataset identifiers PXD027239, PXD027394, and PXD029944.

## Author contributions

ROF and EWT conceived the project. ROF performed all experiments except the following: AG performed validation co-immunoprecipitation and western blot experiments; MS and IPD crystallized HsNMT1:X10-CoA; MMS characterized spectroscopic data. EWT obtained funding and supervised the research. ROF and EWT wrote the manuscript with input from all authors.

## Conflicts of interest

EWT is a founder, shareholder and Director of Myricx Pharma Ltd.

## Supplementary Material

SC-014-D2SC06116C-s001

SC-014-D2SC06116C-s002

SC-014-D2SC06116C-s003

SC-014-D2SC06116C-s004

SC-014-D2SC06116C-s005

SC-014-D2SC06116C-s006
