# Supplementary material for: Discovery of lipid-mediated protein–protein interactions in living cells using metabolic labeling with photoactivatable clickable probes
Source: Chem Sci. 2023 Jan 30;14(9):2419–30. doi: 10.1039/d2sc06116c (PMC9977449; doi:10.1039/d2sc06116c)
Supplement: SC-014-D2SC06116C-s001 [file SC-014-D2SC06116C-s001.pdf]

Electronic Supplementary Material (ESI) for Chemical Science.
This journal is © The Royal Society of Chemistry 2023

Discovery of lipid-mediated protein-protein interactions in living cells using metabolic labeling with photoactivatable clickable probes

*Roman O. Fedoryshchak[1,2], Andrii Gorelik[1,2,3], Mengjie Shen[1,4], Maria M. Shchepinova[1], Inmaculada Pérez-Dorado[1,5], and Edward W. Tate[1,2]**

1. Department of Chemistry, Molecular Sciences Research Hub, 80 Wood Lane, Imperial College London, London W12 0BZ, UK

2. The Francis Crick Institute, 1 Midland Road, London NW1 1AT, UK

3. Current address: Sir William Dunn School of Pathology, University of Oxford, Oxford, OX1 3RE, UK

4. Current address: Charles River Laboratories, 43 Martingale Way, Portishead, Bristol BS20 7AW, UK

5. Current address: Dept. Crystallography & Structural Biology. Institute of Physical-Chemistry Rocasolano - CSIC (Spanish National Research Council). Serrano 119, 28006-Madrid, Spain

* Corresponding author e.tate@imperial.ac.uk

Supplementary information contents

Discovery of lipid-mediated protein-protein interactions in living cells using metabolic labeling with photoactivatable clickable probes 1
Supplementary Figures and Tables 3
Figure S1 3
Figure S2 4
Figure S3 5
Figure S4 7
Figure S5 8
Supplementary Table 3 9
Materials and Methods 10
General chemistry methods 10
Synthesis 10
Biological methods and proteomics 15
Reagents 15
Antibodies 15
Recombinant HsNMT1 and crystallization 15
CPM assay 15
Generation of plasmids 16
Cell culture 17
Metabolic labelling 17
UV crosslinking 17
Cell lysis 17
Protein denaturation 17
Click chemistry (CuAAC ligation) 17
SDS-PAGE and in-gel fluorescence 17
Western blotting 18
Immunoprecipitation-Western blotting 18
Streptavidin enrichment 18
Metabolic labeling and SILAC proteomics 18
UV-crosslinking and SILAC proteomics 19
Label-free interactome proteomics 19
Shotgun proteomics 20
Proteomics data processing (MaxQuant, Perseus and PEAKS) 20
StringDB analysis of protein interactions 20
Full gels corresponding to Figure 1C: 21
Full gels corresponding to Figure 1D: 21
Full Western blots corresponding to Figure 1E: 22
Full Western blots corresponding to Figure 5E 24
Full Western blots corresponding to Figure 5F 26
Quantification of Western blots on Figures 5E, 5F 29
References 30

Supplementary Figures and Tables

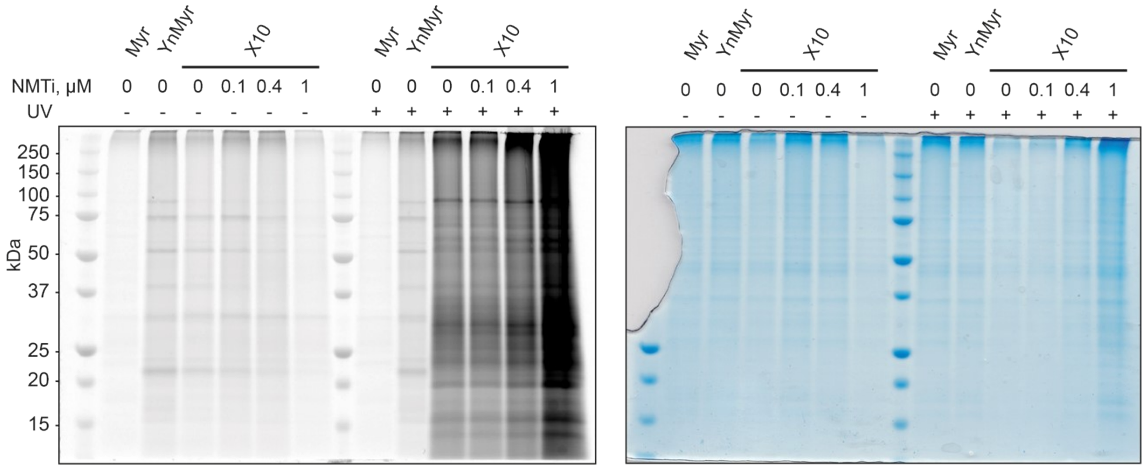

Figure S1. The gel corresponding to Figure 1D in full, covering the whole range of contrast.

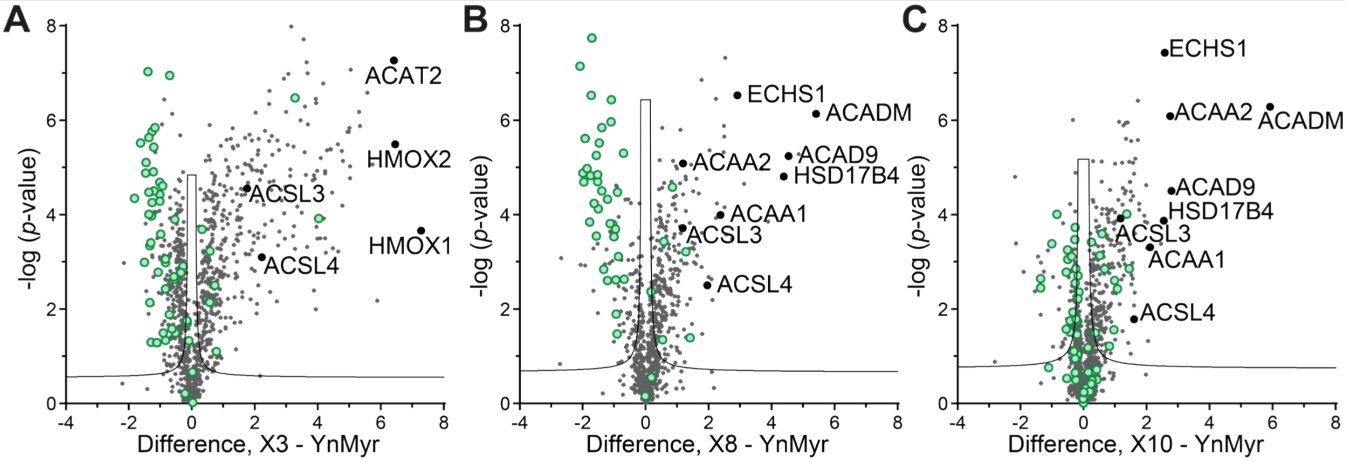

Figure S2. Lipid biosynthesis and metabolism pathway proteins are enriched with probes **X3**/**X8**/**X10**. **A-C**. Samples are in triplicates with relative enrichment plotted on X-axis against statistical significance on Y-axis. Photocrosslinkable clickable probes show differential enrichment of proteins in comparison to **YnMyr**. Fatty acid binding and metabolism proteins are highly enriched with **X3** (**A**), **X8** (**B**) and **X10** (**C**) probes, indicating background levels of non-irradiative crosslinking. **X10** probe shows smaller number and lower enrichment of fatty acid binding proteins. Myristoylated proteins (green circles) are enriched to significantly lower levels with **X3** and **X8** probes than with **YnMyr**. However, with **X10** probe, enrichment of myristoylated proteins is largely similar to **YnMyr**. Selected examples of fatty acid binding and metabolism proteins are indicated. False-discovery rate of 0.05 is shown as a black line.

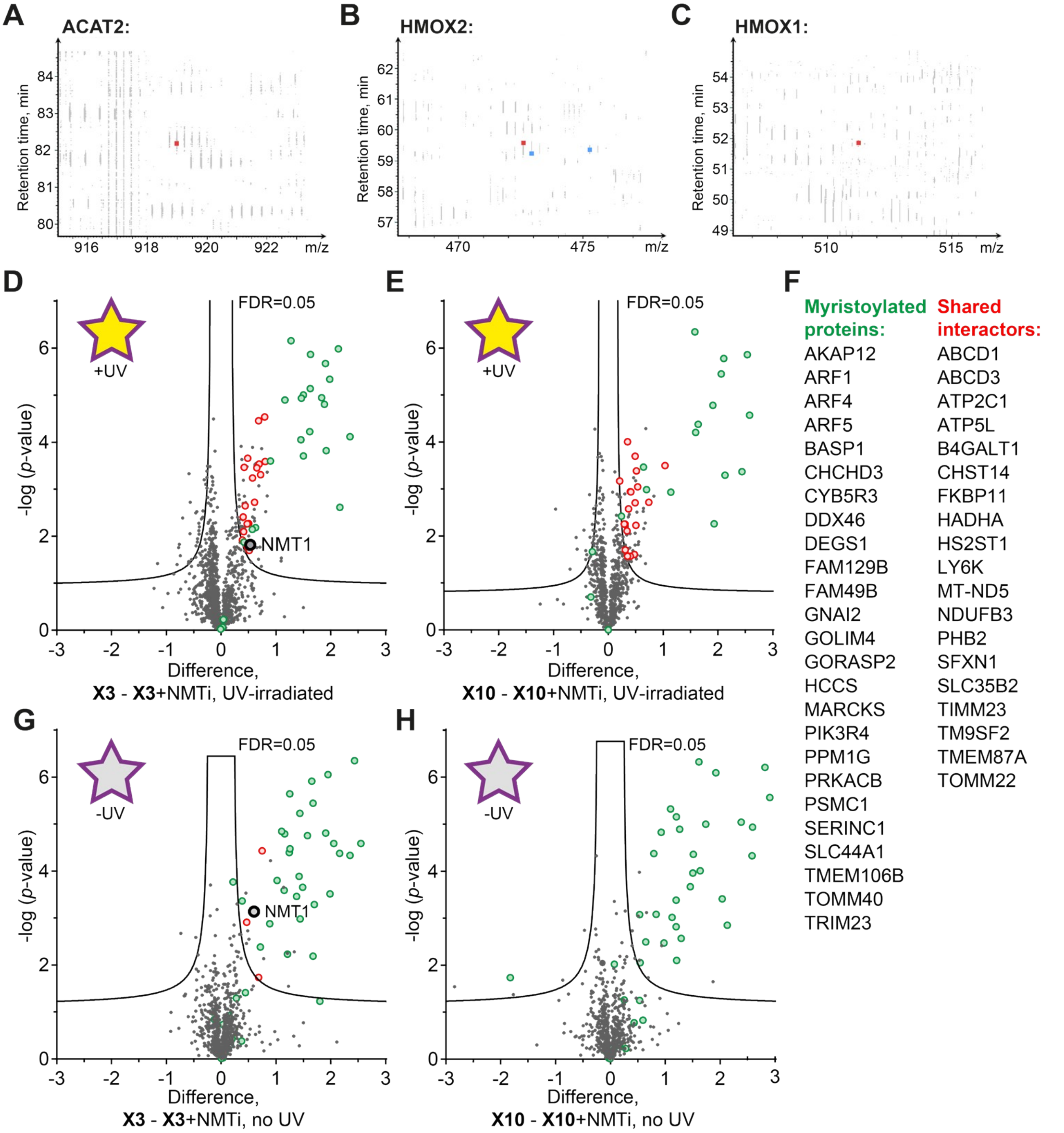

Figure S3. Identification of interactors of myristoylated proteins via photocrosslinking. **A-C**. LCMS elution traces with identified photocrosslinked peptides from ACAT2 (**A**), HMOX2 (**B**) and HMOX1 (**C**) marked in red, other identified peptides are marked in blue. **D-E**. HeLa cells were incubated with 100 µM probes **X3** (**D**) and **X10** (**E**) with or without an NMT inhibitor (NMTi). Cells were lysed in denaturing conditions and processed as shown on Figure 2A, followed by SILAC proteomics. Heavy-labelled spike-in sample was prepared from HeLa cells grown in the SILAC-heavy media and treated with 100 µM of corresponding probes, **X3** or **X10**. Samples are in triplicates with Light/Heavy ratio differences plotted on X-axis against statistical significance on Y-axis. Several myristoylated proteins (green) were enriched compared to cells that were also incubated with 1 µM NMTi. Additionally, several non-myristoylated proteins (shared interactors) appeared as significantly enriched in both **X3** and **X10** experiments (indicated by red circles). **F**. Myristoylated proteins identified in the experiment (left) and proteins that appear significantly enriched in both **X3** and **X10** samples (shared interactors, right). **G-H**. For comparison are the same plots with probes **X3** (**G**) and **X10** (**H**) as on panels **C-D**, but without UV-irradiation. The data is from the SILAC proteomics experiment described in Figure 3A. The enrichment of concomitant proteins is

much lower without UV crosslinking. However, NMT1 appears enriched with **X3** in both UV and no-UV conditions, correlating with results from Western blotting (Figure 2D). Three myristoylated proteins with higher enrichment and/or significance are not shown on panel **E** to preserve the scale.

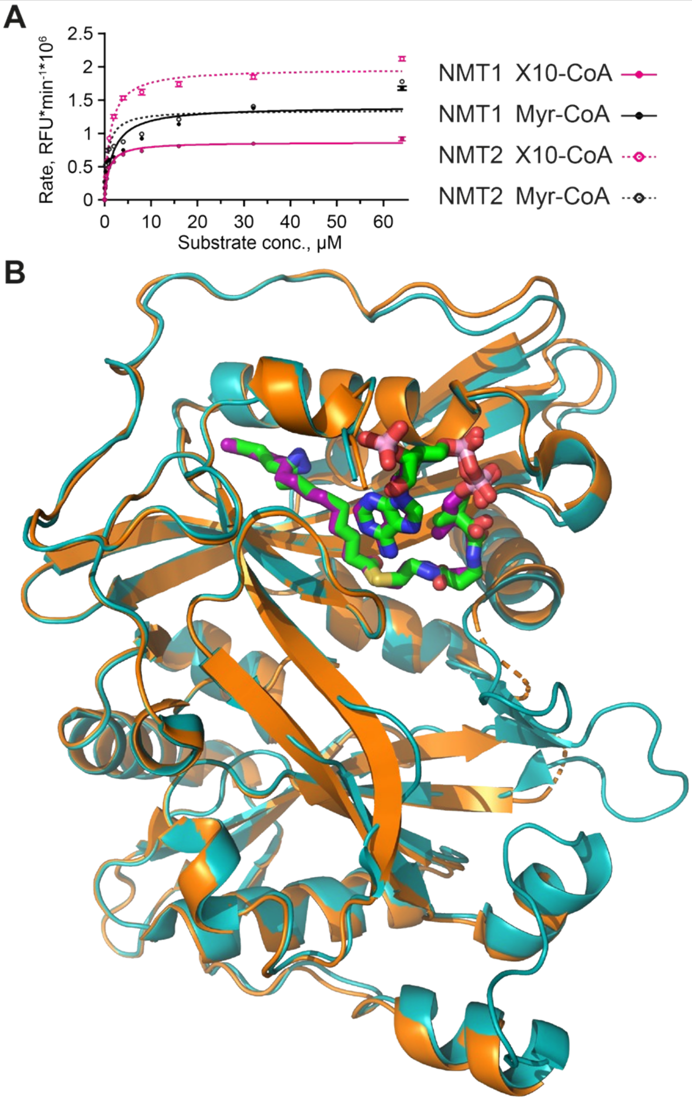

Figure S4. NMT enzymatic assay and crystal structure of HsNMT1:**X10-CoA**. **A**. Rates of enzymatic transfer of **Myr** or **X10** by HsNMT1 or HsNMT2 to a synthetic peptide based on c-Src *N*-terminal peptide (16 µM H-GSNKSKPK-NH2) were measured *in vitro* with CPM-based assay. **B-C**. Structural superimposition of NMT1 (orange) co-crystallized with **X10-CoA** (green) and NMT1 (cyan) co-crystallized with **Myr-CoA** (dark cyan) (PDB:4C2Y). RMSD value between both structures is 0.587 Å for 355 Cα atoms. Whole protein view (**B**) and close-up of the myristate pocket (**C**). **D**. 2Fo-Fc electron density map observed for **X10-CoA** (chain A) contoured at sigma=1.0. Color code used is blue (nitrogen), red (oxygen), yellow (sulfur), and pink (phosphorus).

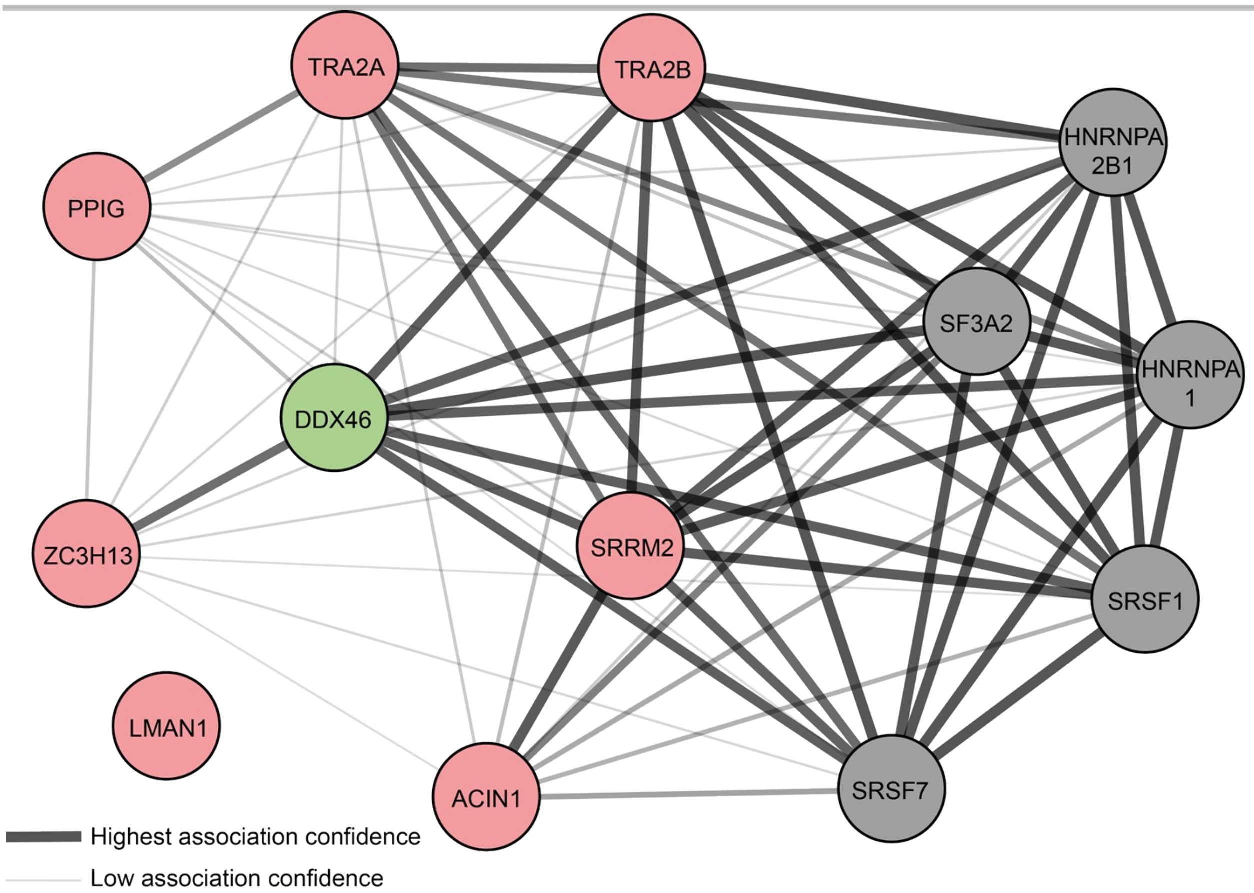

Figure S5. Association network of DDX46 (green) and its interactors identified in this work (red) based on analysis with StringDB (1). Proteins that are high-confidence interactors that were not identified in our experiments are shown in grey. Lines indicate known protein associations.

Supplementary Table 3. X-ray data collection and refinement statistics.

	HsNMT1-X10
Data collection statistics	
Space group	$P2_12_12$
Number of complexes in ASU	2
Unit cell (Å)	*a*=79.97<br>*b*=177.45<br>*c*=58.24
Wavelength (Å)	0.97625
Beamline	I03 (DIAMOND)
Resolution range (Å)	177.46-2.37 (2.41-2.37)
R_{merge}	0.191 (0.847)
R_{pim}	0.076 (0.353)
Number of total reflections	247356 (11404)
Number of unique reflections	34474 (1709)
Mean ([I]/SD[I])	9.3 (2.2)
$CC_{1/2}$	0.994 (0.661)
Completeness (%)	100 (99.7)
Multiplicity	7.2 (6.7)
Wilson plot B (Å2)	32.66
Refinement	
Number of atoms	6174
Protein	5889
X10	130
Ions / Glycerol	2 Mg^{2+}, 24
Water	129
R_{work} (%)	19.9
R_{free} (%)	26.1
<B > (Å^2)	34.61
rms bond length deviation (Å)	0.008
rmsd angle deviation (°)	1.037

Values in parentheses correspond to the highest resolution shell.

$R_{merge} = \Sigma(I_{hl} - < I_h >)/\Sigma < I_h >$

$R_{meas} = \Sigma\sqrt{}(n_h/n_h - 1)(I_{hl} - < I_h >)/\Sigma < I_h >$

$R_{pim} = \Sigma\sqrt{}(1/n_h - 1)(I_{hl} - < I_h >)/\Sigma < I_h >$

Materials and Methods

General chemistry methods

All chemical reagents and solvents were purchased from commercial sources and used as supplied without any further purification. Dry solvents were dispensed using Pure SolvTM solvent drying towers (Innovative technology Inc.) or purchased anhydrous from commercial sources. All anhydrous conditions were performed in oven-dried glassware under argon atmosphere. For purifications, HPLC-grade solvents (≥ 99% purity) were used (Merck or Fisher Scientific UK). Room temperature (RT) reactions were carried out at 15-20°C. The term *in vacuo* refers to solvent removal using Büchi rotary evaporation in a water bath at 15-50°C, at ~ 10 mm Hg.

Reactions were monitored by thin-layer chromatography (TLC), using TLC plates pre-coated with silica gel 60 F_{254} on aluminium (Merck). TLC plates were visualized using UV light (254 nm) or by using the appropriate chemical stain. Flash column chromatography was carried out using Geduran® Si 60 (40-63 µm) silica gel (Merck). The purification and analysis of conjugate **X10-CoA** was performed on a Waters RP-HPLC system (Waters 2767 autosampler; Waters 515 HPLC pump; Waters 3100 mass spectrometer with ESI; Waters 2998 photodiode array (detection from 190 to 700 nm)), equipped with Xbridge C18 reverse-phase columns with dimensions 4.6 mm × 100 mm for analytical (1.2 mL/min flow rate) and 19 mm × 100 mm for preparative (20 mL/min flow rate) runs.

Nuclear magnetic resonance (NMR) spectra were recorded at ambient temperature on a Bruker AV400 instrument operating at 400 MHz for ^{1}H and 101 MHz for ^{13}C in the deuterated chloroform $CDCl_3$ (δ_H = 7.26 and δ_C = 77.16) as the internal standard. Chemical shifts (δ) are reported in parts per million (ppm) relative to tetramethylsilance (TMS) as a reference and coupling constants (*J*) in Hertz (Hz). The multiplicity of each signal is indicated as s-singlet, d-doublet, t-triplet, q-quartet, quint-quintet, m-multiplet or a combination of these. All assignments were made with the help of COSY, HSQC, HMBC, NOESY or DEPT correlation spectra. Spectra were processed and analysed using MestReNova© 12 NMR software. High-resolution mass spectra (HRMS) were recorded on a Waters LCT Premier mass spectrometer, operating in electrospray ionization (ESI) (positive (+) and negative (-)) mode at the Department of Chemistry, Imperial College London. The *m/z* values are reported in Daltons.

Synthesis

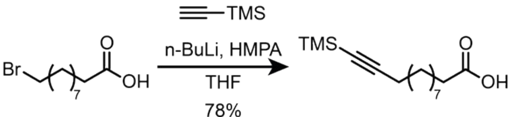

1 2

Preparation of 12-(trimethylsilyl)dodec-11-ynoic acid (2). The procedure is modified from (2). Trimethylsilylacetylene (608 µL, 4.4 mmol) in dry THF (12 mL) was cooled to -78°C in dry ice-acetone bath. A solution of n-BuLi in hexane (2.5 M, 1.76 mL, 4.4 mmol) was added dropwise under argon and stirred for 20 min. 10-Bromodecanoic acid **1** (502 mg, 2.0 mmol) was dissolved in dry THF (4 mL) and added to the reaction mixture followed by addition of hexamethylphosphoramide (HMPA) (4 mL, 22 mmol). The solution was stirred overnight and allowed to warm up to RT. The reaction mixture was quenched with saturated $NH_4Cl_{(aq.)}$, the pH was adjusted to 3 (with 1 M HCl). The product was extracted with EtOAc and washed with brine. The organic phase was dried over Na_2SO_4 (anh.) and concentrated *in vacuo*. The crude residue was purified by flash chromatography eluting with 12:87:1 EtOAc:Hexane:AcOH mixture, to give acid **2** (330 mg, 62%).^{1}H NMR (400 MHz, $CDCl_3$): δ 2.35 (t, *J* = 7.5 Hz, 2H), 2.21 (t, *J* = 7.1 Hz, 2H), 1.63 (quint, *J* = 7.5 Hz, 2H), 1.50 (quint, *J* = 7.2 Hz, 2H), 1.41-1.23 (m, 10H), 0.14 (s, 9H). ^{13}C NMR (101 MHz, $CDCl_3$): δ 179.68, 107.88, 84.42, 34.08, 29.40, 29.31, 29.17, 29.15, 28.90, 28.75, 24.81, 19.99, 0.33.

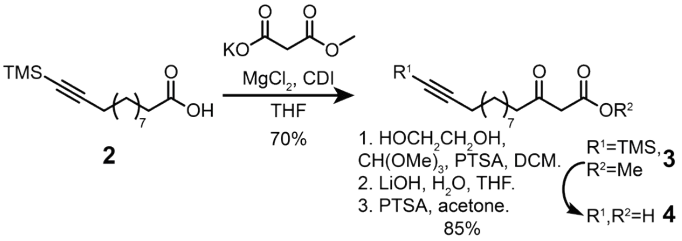

Preparation of methyl 3-oxo-14-(trimethylsilyl)tetradec-13-ynoate (3). The procedure is modified from (3). A suspension of $MgCl_2$ (1.15 g, 7.6 mmol) and methyl potassium malonate (2.48 g, 15.9 mmol) in dry THF (20 mL) was stirred at 50°C for 4 h. Separately, carbonyldiimidazole (CDI) (1.47 g, 9.1 mmol) was added to the solution of acid **2** (2.03 g, 7.6 mmol) in dry THF (20 mL) in 4°C ice bath. The mixture was allowed to warm up to RT, stirred for 1 h and then added dropwise to methyl magnesium malonate suspension. The resulting mixture was stirred overnight, then concentrated *in vacuo* and resuspended in EtOAc. The solution was washed with 1 M $KHSO_{4\,(aq.)}$, $NaHCO_{3\,(aq.)}$ and brine. The organic phase was dried over Na_2SO_4 (anh.) and concentrated *in vacuo*. The crude residue was purified by flash chromatography eluting with 1:4 EtOAc:Hexane mixture, to give β-keto ester **3** (1.968 g, 80%). ^{1}H NMR (400 MHz, $CDCl_3$): δ 3.73 (s, 3H), 3.44 (s, 2H), 2.52 (t, *J* = 7.5 Hz, 2H), 2.19 (t, *J* = 7.2 Hz, 2H), 1.58 (quint, *J* = 7.5 Hz, 2H), 1.49 (quint, *J* = 7.3 Hz, 2H), 1.40-1.22 (m, 10H), 0.13 (s, 9H). ^{13}C NMR (101 MHz, $CDCl_3$): δ 202.94, 167.83, 107.85, 84.39, 52.45, 49.15, 43.20, 29.40, 29.38, 29.13, 29.10, 28.88, 28.73, 23.57, 19.97, 0.31. HRMS (ESI$^+$, m/z): calcd. for $C_{18}H_{32}O_3NaSi$ [M+Na]$^+$ 347.2018; found 347.2033.

Preparation of 3-oxotetradec-13-ynoic acid (4). β-Keto ester **3** (1.47 g, 4.5 mmol) was dissolved in DCM (15 mL), followed by addition of ethylene glycol (1.41 g, 22.7 mmol), *p*-toluenesulfonic acid hydrate (PTSA) (86 mg, 0.45 mmol) and trimethyl orthoformate (1.01 g, 9.5 mmol). The mixture was refluxed overnight and quenched with saturated $NaHCO_{3\,(aq.)}$ (15 mL). The crude product was extracted with DCM and washed with brine. The organic phase was dried over Na_2SO_4 (anh.) and concentrated *in vacuo* to obtain a glycol-protected intermediate **3'** in quantitative yield (1.65 g, 100%), that was used without further purification.

A glycol-protected intermediate **3'** (1.65 g, 4.5 mmol) was dissolved in THF (54 mL), 1 M $LiOH_{(aq.)}$ (18 mL) was added and the mixture was stirred at 60°C for 4 h. The pH was then adjusted to 3 (with 1 M HCl). The crude product was extracted with DCM and washed with brine. The organic phase was dried over Na_2SO_4 (anh.) and concentrated *in vacuo* to obtain acid intermediate **3''** (1.17 g, 92%).

The acid intermediate **3''** (402 mg, 1.43 mmol) was dissolved in acetone (5 mL), PTSA (68 mg, 0.36 mmol) was added, and the mixture was stirred for 2 h at RT. EtOAc was added, and the solution was washed with H_2O and brine. The organic phase was dried over Na_2SO_4 (anh.) and concentrated *in vacuo*. The crude residue was purified by flash chromatography eluting with 20:80:1 EtOAc:Hexane:AcOH mixture, to yield acid **4** (317 mg, 93 %). ^{1}H NMR (400 MHz, $CDCl_3$): δ 3.52 (s, 2H), 2.56 (t, *J* = 7.5 Hz, 2H), 2.18 (td, *J* = 7.1, 2.7 Hz, 2H), 1.94 (t, *J* = 2.6 Hz, 1H), 1.67-1.57 (m, 2H), 1.57-1.47 (m, 2H), 1.43-1.22 (m, 10H). HRMS (ESI$^-$, m/z): calcd. for $C_{14}H_{21}O_3$ [M-H]$^-$ 237.1491; found 237.1500.

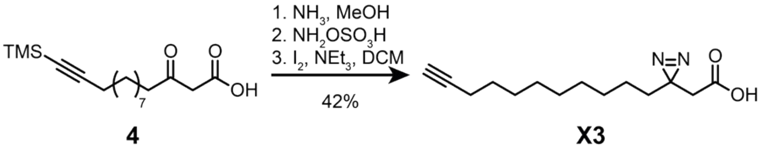

Preparation of 2-(3-(undec-10-yn-1-yl)-3H-diazirin-3-yl)acetic acid (X3). The procedure is modified from (4). Concentrated $NH_{3\,(aq.)}$ was boiled in a 3-neck flask and carried under the flow of argon through a gas trap with NaOH pellets. Liquid NH_3 (~ 5 mL) was collected into a 3-neck flask equipped with a dry ice-acetone condenser at -78°C. Acid **4** (150 mg, 0.75 mmol) was dissolved in dry MeOH (5 mL) and added to the liquid NH_3. The mixture was kept stirring at -30 to -40°C for 3 h by addition of dry ice. Then, hydroxylamine-*O*-sulfonic acid (94 mg, 0.83 mmol) was dissolved in dry MeOH (2 mL) and added to the

reaction mixture over 30 min at -78°C in dry ice-acetone bath. The reaction was left to warm up to RT overnight. Next, argon was bubbled through the solution for 1 h to volatilize leftover NH_3. The solution was filtered through sintered glass, and the residue was washed twice with dry MeOH. The filtrate was concentrated *in vacuo* at RT and the residue was cooled on ice and dissolved in DCM (5 mL), followed by NEt_3 (220 µL, 2.25 mmol). Iodine (~1 g) solution in DCM (10 mL) was added dropwise until brown color persisted and the mixture was left stirring at RT for 1 h. The solution was then washed with 1 M HCl (5 mL), 1 M $Na_2S_2O_{3\,(aq.)}$ (5 mL) and brine (5 mL). The organic phase was dried over Na_2SO_4 (anh.) and concentrated *in vacuo*. The crude residue was purified by flash chromatography eluting with 20:80:1 EtOAc:Hexane:AcOH mixture, yielding diazirine **X3** as a white powder (66 mg, 42%). ^{1}H NMR (400 MHz, $CDCl_3$): δ 2.30 (s, 2H), 2.17 (td, *J* = 7.1, 2.7 Hz, 2H), 1.94 (t, *J* = 2.6 Hz, 1H), 1.59-1.47 (m, 4H), 1.43-1.32 (m, 2H), 1.31-1.18 (m, 8H), 1.14-1.03 (m, 2H). ^{13}C NMR (101 MHz, $CDCl_3$): δ 175.20, 84.91, 68.22, 39.62, 32.53, 29.41, 29.38, 29.14 (two overlapping peaks), 28.83, 28.59, 26.02, 23.86, 18.53. HRMS (ESI^-, m/z): calcd. for $C_{14}H_{21}N_2O_2$ $[M-H]^-$ 249.1603; found 249.1597. NMR purity 99%.

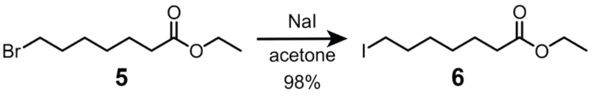

Preparation of ethyl 7-iodoheptanoate (6). To a clear solution of ethyl 7-bromoheptanoate **5** (6.08 g, 25.7 mmol) in acetone (50 mL) was added NaI (11.54 g, 77 mmol). The mixture was stirred at RT overnight. Completion of the reaction was checked by ^{1}H NMR. After full conversion, the mixture was filtered through sintered glass and concentrated *in vacuo,* followed by addition of H_2O (100 mL) and DCM (100 mL). The layers were separated, and the aqueous layer was extracted again with DCM (100 mL). The combined organic layers were washed with 1 M $Na_2S_2O_{3\,(aq.)}$ (50 mL), brine (50 mL), dried over Na_2SO_4 (anh.) and concentrated *in vacuo,* yielding the crude iodide **6** (7.1 g, 98%), that was used without further purification. ^{1}H NMR (400 MHz, $CDCl_3$): δ 4.12 (q, *J* = 7.1 Hz, 2H), 3.18 (t, *J* = 7.0 Hz, 2H), 2.29 (t, *J* = 7.5 Hz, 2H), 1.82 (quint, *J* = 7.0 Hz, 2H), 1.63 (quint, *J* = 7.3 Hz, 2H), 1.48-1.30 (m, 4H), 1.25 (t, *J* = 7.1 Hz, 3H).

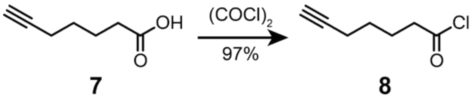

Preparation of hept-6-ynoyl chloride (8). The procedure is modified from (5). Oxalyl chloride solution in DCM (2 M, 17.5 mL, 35 mmol) was mixed with a solution of 6-pentynoic acid **7** (0.63 g, 5 mmol) in DCM (50 mL) under argon, the mixture was heated to 70˚C, refluxed for 1 h and stirred overnight at RT. The volatiles were evaporated *in vacuo*, yielding the crude chloride **8** in quantitative yield (0.72 g), that was used without further purification. ^{1}H NMR (400 MHz, $CDCl_3$): δ 2.93 (t, *J* = 7.2 Hz, 2H), 2.23 (td, *J* = 7.0, 2.7 Hz, 2H), 1.97 (t, *J* = 2.6 Hz, 1H), 1.89-1.80 (m, 2H), 1.64-1.55 (m, 2H).

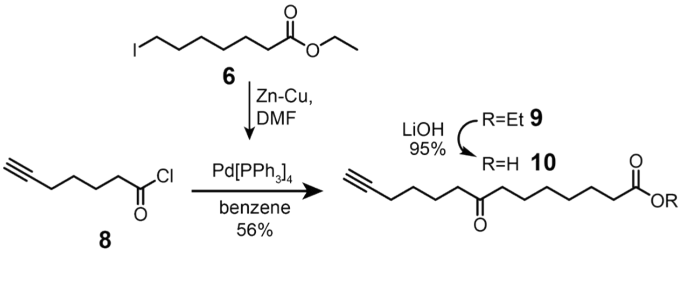

Preparation of ethyl 8-oxotetradec-13-ynoate (9). The procedure is modified from (6). Zn-Cu powder (1-3% Cu, 392 mg, 6 mmol) was dried at 150°C *in vacuo*. Next, iodide **6** (1.42 g, 5 mmol), dry benzene (20 mL) and DMF (0.5 mL, 6.5 mmol) were mixed under argon at RT, the solution was heated to 80°C and stirred for 5 h to form alkylzinc iodide. In the second flask, chloride **8** (0.65 g, 4.5 mmol) and freshly prepared $Pd[PPh_3]_4$ (7) (174 mg, 0.15 mmol) were mixed and dissolved in dry benzene (15 mL) under argon. The solution of alkylzinc iodide was cooled down to RT and added to the second flask via a syringe pump over the course of 40 min and left to stir overnight at RT. The reaction was quenched

with saturated NH_4Cl (aq.) (50 mL), followed by addition of EtOAc (50 mL). The layers were separated, and the aqueous layer was extracted again with EtOAc (50 mL). The combined organic phases were washed with brine, dried over Na_2SO_4 (anh.) and concentrated *in vacuo*. The crude residue was purified by flash chromatography eluting with a three-step gradient of 7-10-12% of EtOAc in hexane, obtaining ethyl ester **9** as brown transparent crystals (673 mg, 57%). ^{1}H NMR (400 MHz, $CDCl_3$): δ 4.10 (q, *J* = 7.2 Hz, 2H), 2.42-2.35 (m, 4H), 2.26 (t, *J* = 7.5 Hz, 2H), 2.18 (td, *J* = 7.0, 2.6 Hz, 2H), 1.93 (t, *J* = 2.7 Hz, 1H), 1.71-1.45 (m, 8H), 1.33-1.26 (m, 4H), 1.23 (t, *J* = 7.1 Hz, 3H). HRMS (ESI$^+$, m/z): calcd. for $C_{16}H_{27}O_3$ $[M+H]^+$ 267.1960; found 267.1973.

Preparation of 8-oxotetradec-13-ynoic acid (10). Ethyl ester **9** (1.36 g, 5.1 mmol) was dissolved in THF (45 mL), followed by 1 M LiOH (aq.) (15 mL) and the mixture was stirred overnight at RT. Next, the pH was adjusted to 2 (with 1 M HCl). The crude product was extracted with EtOAc (×3) and washed with brine. The organic phase was dried over Na_2SO_4 (anh.) and concentrated *in vacuo*. The crude residue was purified by flash chromatography eluting with 20:80:1 EtOAc:Hexane:AcOH mixture, yielding acid **10** (1.15 g, 95%). ^{1}H NMR (400 MHz, $CDCl_3$): δ 2.44-2.37 (m, 4H), 2.34 (t, *J* = 7.5 Hz, 2H), 2.19 (td, *J* = 7.0, 2.7 Hz, 2H), 1.94 (t, *J* = 2.6 Hz, 1H), 1.74-1.47 (m, 8H), 1.39-1.23 (m, 4H). HRMS (ESI$^+$, m/z): calcd. for $C_{14}H_{23}O_3$ $[M+H]^+$ 239.1647; found 239.1644.

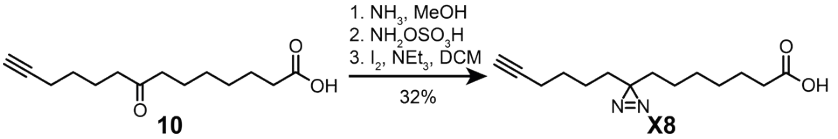

Preparation of 7-(3-(hex-5-yn-1-yl)-3H-diazirin-3-yl)heptanoic acid (X8). Diazirine **X8** was synthesized following the same procedure as described for diazirine **X3** (see above) starting with acid **10** (196 mg, 0.82 mmol). The crude residue was purified using flash chromatography eluting with 15:85:1 EtOAc:Hexane:AcOH mixture, yielding diazirine **X8** as a white powder (63 mg, 32%). ^{1}H NMR (400 MHz, $CDCl_3$): δ 2.33 (t, *J* = 7.4 Hz, 2H), 2.14 (td, *J* = 7.0, 2.7 Hz, 2H), 1.94 (t, *J* = 2.7 Hz, 1H), 1.60 (quint, *J* = 7.4 Hz, 2H), 1.46 (quint, *J* = 7.4 Hz, 2H), 1.39-1.32 (m, 4H), 1.32-1.15 (m, 6H), 1.12-1.03 (m, 2H). ^{13}C NMR (101 MHz, $CDCl_3$): δ 180.24, 84.11, 68.68, 34.07, 32.85, 32.57, 28.91, 28.90, 28.73, 28.02, 24.58, 23.75, 23.10, 18.32. HRMS (ESI$^+$, m/z): calcd. for $C_{14}H_{23}N_2O_2$ $[M+H]^+$ 251.1760; found 251.1758. NMR purity 99%.

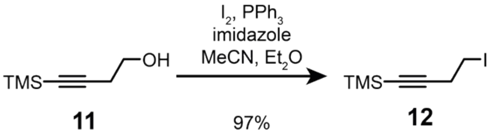

Preparation of (4-iodobut-1-yn-1-yl)trimethylsilane (12). 4-(Trimethylsilyl)but-3-yn-1-ol **11** (2.44 mL, 15 mmol) was dissolved in the mixture of MeCN (20 mL) and Et_2O (60 mL). Triphenylphosphine (5.25 g, 20 mmol), imidazole (1.36 g, 20 mmol) and iodine (5.08 g, 20 mmol) were added to the solution. The reaction mixture was stirred for 2 h at RT, followed by quenching with saturated $NaHCO_3$ (aq.). The organic phase was washed with 1 M $Na_2S_2O_3$ (aq.), brine and dried over Na_2SO_4 (anh.). The solution was passed through a layer of silica on a glass filter and washed off with hexane. The filtrate was concentrated *in vacuo* to obtain iodide **12** (3.67 g, 97%) that was used without further purification. ^{1}H NMR (400 MHz, $CDCl_3$): δ 3.22 (t, *J* = 7.5 Hz, 2H), 2.79 (t, *J* = 7.5, 2H), 0.16 (s, 9H).

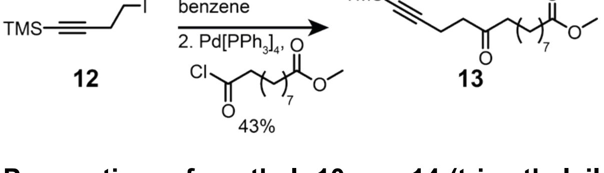

Preparation of methyl 10-oxo-14-(trimethylsilyl)tetradec-13-ynoate (13). Ethyl ester **13** was synthesized following the same procedure as described for the ester **9** (see above) starting with iodide **12** (2.52 g, 10 mmol) and methyl 10-chloro-10-oxodecanoate (1.91 g, 8.1 mmol). The crude residue

was purified using flash chromatography eluting with 5:95 EtOAc:Hexane mixture, yielding ethyl ester **13** (1.25 g, 43%). The product was ~78% pure due to the methyl 10-chloro-10-oxodecanoate contamination (calculated by ^{1}H NMR). ^{1}H NMR (400 MHz, $CDCl_3$): δ 3.65 (s, 3H), 2.65-2.59 (m, 2H), 2.49-2.43 (m, 2H), 2.40 (t, *J* = 7.4 Hz, 2H), 2.28 (t, *J* = 7.5 Hz, 2H), 1.64-1.51 (m, 4H), 1.32-1.22 (m, 8H), 0.11 (s, 9H).

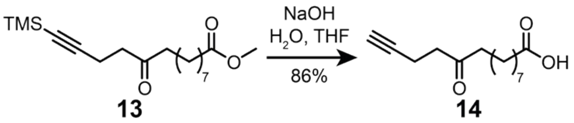

Preparation of 10-oxotetradec-13-ynoic acid (14). Acid **14** was synthesized following the same procedure as described for the acid **10** (see above) starting with ethyl ester **13** (1.25 g, 4.4 mmol). The crude residue was purified using flash chromatography eluting with 20:80:1 EtOAc:Hexane:AcOH mixture, yielding acid **14** (0.79 g, 86%). ^{1}H NMR (400 MHz, $CDCl_3$): δ 2.68-2.63 (m, 2H), 2.47-2.39 (m, 4H), 2.33 (t, *J* = 7.5 Hz, 2H), 1.94 (t, *J* = 2.7 Hz, 1H), 1.67-1.53 (m, 4H), 1.36-1.23 (m, 8H).

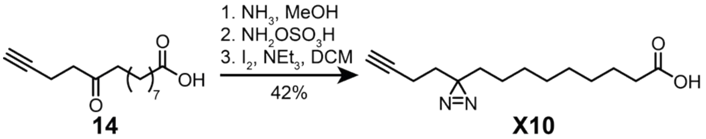

Preparation of 9-(3-(but-3-yn-1-yl)-3H-diazirin-3-yl)nonanoic acid (X10). Diazirine **X10** was synthesized following the same procedure as described for diazirine **X3** (see above) starting with acid **14** (150 mg, 0.63 mmol). The crude residue was purified using flash chromatography eluting with 15:85:1 EtOAc:Hexane:AcOH mixture, yielding diazirine **X10** as a white powder (67 mg, 42%). ^{1}H NMR (400 MHz, $CDCl_3$): δ 2.34 (t, *J* = 7.5 Hz, 2H), 2.02-1.96 (m, 3H), 1.65-1.58 (m, 4H), 1.44-1.38 (m, 2H), 1.36-1.19 (m, 8H), 1.13-1.02 (m, 2H). ^{13}C NMR (101 MHz, $CDCl_3$): δ 179.73, 83.03, 69.13, 34.06, 32.75, 32.55, 29.27, 29.20, 29.18, 29.09, 28.39, 24.75, 23.89, 13.48. HRMS (ESI$^-$, m/z): calcd. for $C_{14}H_{21}N_2O_2$ $[M-H]^-$ 249.1603; found 249.1593. NMR purity 99%.

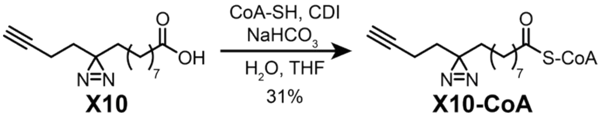

Preparation of X10-CoA conjugate. X10-CoA conjugate was synthesized using the procedure described in (8). Diazirine **X10** (16 mg, 0.064 mmol) was dissolved in dry THF (1.0 mL) under nitrogen. Solution of CDI (10 mg, 0.064 mmol) in DCM (0.5 mL) was added and reaction mixture was stirred at RT for 30 min. The mixture was concentrated *in vacuo* and resuspended in dry THF (2.0 mL). CoA-SH (25 mg, 0.032 mmol) was dissolved in $NaHCO_{3\ (aq.)}$ (0.5 M, 2.0 mL) and added to the reaction mixture. The reaction was stirred for 4 h at RT under argon. THF was then removed *in vacuo* and the remaining aqueous phase was purified by preparative RP-HPLC. For preparative RP-HPLC a gradient of MeCN in 10 mM NH_4OAc pH 5.2 (0–5 min 0% MeCN, 5–10 min linear gradient 0-50% MeCN, 10–15 min 50% MeCN) was used. The collected fractions were combined and concentrated *in vacuo* to give **X10-CoA** (21 mg, 31%) as a white solid. HRMS (ESI$^-$, m/z): calcd. for $C_{35}H_{55}N_9O_{17}SP_3$ $[M-H]^-$ 998.2650; found 998.2640.

Biological methods and proteomics

Reagents

All reagents were purchased from commercial sources and used as supplied. Human embryonic kidney 293T cells (HEK293T) and HeLa cells were obtained from ATCC and verified by STR by The Francis Crick Cell Services. MyrCoA, YnMyr, HsNMT1, HsNMT2, Hs pp60src(2–9) N-terminal peptide H-GSNKSKPK-NH_2 were available from the laboratory and were described previously (9,10). Click reagents AzTB (azido-TAMRA-biotin used for enrichment on the streptavidin beads followed by in-gel fluorescence analysis or Western blotting), AzT (azido-TAMRA used for in-gel fluorescence analysis) and AzRB (azido-arginine-biotin used for enrichment on neutravidin beads and proteomics analysis) were available from the laboratory, their syntheses have been described previously (11). Ultrapure water was obtained on a MilliQ Millipore water purification system.

Antibodies

DDX46 (Atlas antibodies, HPA036554; rabbit, 1:1000 dilution), FSP1 (Proteintech, 20886-1-AP, rabbit, 1:1000 dilution), GAPDH (Proteintech, 60004-1-Ig; mouse, 1:1000 dilution), NMT1 (Atlas antibodies, HPA022963; rabbit, 1:500 dilution), PSMC1 (Atlas antibodies, HPA000872; rabbit, 1:500 dilution), TOMM40 (Proteintech, 18409-1-AP; rabbit, 1:1000 dilution), Tra2β (Abcam, ab31353; rabbit, 1:1000 dilution), tubulin (Merck, T5168; mouse, 1:5000 dilution).

Recombinant HsNMT1 and crystallization

HsNMT1 was produced as a 6-His-tagged protein as described (9) and was available in the laboratory. HsNMT1 was crystallized in complex with **X10-CoA** as previously described for HsNMT1:**Myr-CoA** complex using the sitting-drop vapor diffusion method at 20˚C (12). Crystals of HsNMT1 in complex with **X10-CoA** we obtained in 2 µL droplets formed by mixing equal volumes of a protein solution at 7.05 mg/mL (supplemented with 0.5 mM of **X10-CoA** and 5% DMSO) and a cryosolution containing the precipitant components (16-20% (v/w) PEG 4K, 5 mM Ni(**II**) chloride, 100 mM sodium citrate pH 4.5, and 2.5% (v/v) glycerol). Crystals were then flashed cooled in liquid nitrogen and stored for data collection. A complete X-ray data set at 2.37 Å resolution was collected at 100*K* and using synchrotron radiation at Diamond Light Source (in Oxford, UK). The data set was integrated with XDS (13), and scaled and reduced using AIMLESS (14) from CCP4 package. Crystals belong to the $P2_12_12$ space group and have unit cell dimensions a=79.97 Å, b=177.45 Å, c=58.24 Å. Structure resolution was accomplished using the molecular replacement method with the software PHASER(15) and the crystallographic structure of HsNMT1:**Myr-CoA** complex as input model (PDB entry 4C2Y(12)). The initial model was subjected to alternate cycles of model building using COOT (16) and refinement using REFMAC-5 (17) and PHENIX 1.10.1 (18). The structure consists of two molecules of HsNMT1:**X10-CoA** complex per asymmetric unit (chains A and B), and thus it was refined using NCS restrictions. Overall, the resulting electron density allowed to modeled almost entirely both NMT1 molecules, with the exception of 9 N-terminal residues of both A and B chains (NMT residues 109-114 and 3 more residues of the N-terminal tag), residues 182-188, 308-319, 407-414 of chain A, and residues 312-318 of chain B, which were flexible and thus not visible on the map. Electron density maps of good quality were obtained for the **X10-CoA** in both molecules of complex (Figure S1B). The cif file for the refinement of the **X10-CoA** probe was generated with ELBOW (19) as part of the PHENIX package. Difference electron density maps were calculated in PHENIX. The geometry of the final models was validated using MOLPROBITY (20). Figures were generated using PYMOL (DeLano Scientific LLC, http://pymol.sourceforge.net/). X-ray data collection and statistics are summarized in Table 1.

CPM assay

The assay was performed as previously described in (10). Briefly, various concentrations of either **Myr-CoA** or **X10-CoA** were added to hNMT1 or hNMT2 (200 ng/mL), peptide substrate (16 µM Hs $pp60^{src}$(2–9) N-terminal peptide H-Gly-Ser-Asn-Lys-Ser-Lys-Pro-Lys-NH2) and 7-diethylamino-3-(4′-maleimidylphenyl)-4-methylcoumarin (8 µM) on a 96-well plate, 4 replicates per condition. Accumulation of fluorescence signal at 470 nm was measured at one-minute intervals during the first 15 min of the reaction. Rates of the enzymatic reaction were calculated based on the plotted linear range of fluorescence signal over time using GraphPad Prizm 5. The pseudo first order dependence of enzymatic rates on substrate concentration were fitted using Michaelis-Menten equation.

Generation of plasmids

mCitrine-TST (twin-strep-tag), Src-TST and Src6Q-TST constructs in pCMV vector were a kind gift from A. Konitsiotis.

Other TST-tagged constructs were assembled using NEBuilder® HIFI DNA Assembly Master Mix (New England Biolabs). The vector was PCR amplified (primers FW: TGGAGCCATCCGCAGTTTG; REV: CATGGTGGCGAAGCTTGAG). Genes of interest were amplified from HEK293T cells cDNA or plasmids available in the laboratory using the following primers:

RP2 FW: gctcaagcttcgccaccatgGGCTGCTTCTTC;
RP2 REV: tcaaactgcggatggctccaTATTCCCATCTGTATATCAGCAAAG;
FAM49B FW: gctcaagcttcgccaccatgGGGAATCTTCTTAAAGTTTTG;
FAM49B REV: tcaaactgcggatggctccaTTGCAGCATGGATTTAATTTG;
DDX46 FW: GGTACTCAAGGGCACCAGTATTC;
DDX46 REV: CCACATACATCATAGACCAGCAC;
NOL3 FW: gctcaagcttcgccaccatgGGCAACGCGCAGGAG;
NOL3 REV tcaaactgcggatggctccaGGAATCTTCGGACTCGTCCCTTTC;
GNAI2 FW gctcaagcttcgccaccatgGGCTGCACCGTGAG;
GNAI2 REV tcaaactgcggatggctccaGAAGAGGCCGCAGTCC;
GNAI3 FW gctcaagcttcgccaccatgGGCTGCAC;
GNAI3 REV tcaaactgcggatggctccaATAAAGTCCACATTCCTTTAAGTTG;
FSP1 FW gctcaagcttcgccaccatgGGGTCCCAGGTCTC;
FSP1 REV tcaaactgcggatggctccaAGGTGGAGACTGCCTCATG;
NCS1 FW gctcaagcttcgccaccatgGGGAAATCCAACAGCAAG;
NCS1 REV tcaaactgcggatggctccaTACCAGCCCGTCGTAGAG;
BASP1 FW gctcaagcttcgccaccatgGGAGGCAAGCTCAG;
BASP REV tcaaactgcggatggctccaCTCTTTCACGGTTACGGTTTG;
ARF1 FW: gctcaagcttcgccaccatgGGGAACATCTTCGCCAAC;
ARF1 REV: tcaaactgcggatggctccaCTTCTGGTTCCGGAGCTG;
CHP1 FW: gctcaagcttcgccaccatgGGTTCTCGGGCCTC;
CHP1 REV: tcaaactgcggatggctccaGTGAAGAAATCGGATGCTCATTTTCTG;
GNAO1 FW: gctcaagcttcgccaccatgGGATGTAC;
GNAO1 REV: tcaaactgcggatggctccaGTACAAGCCGCAGC;
PSMC1 FW: gctcaagcttcgccaccatgGGTCAAAGTCAGAGTG;
PSMC1 REV: tcaaactgcggatggctccaGAGATACAGCCCCTCAG;
ABL1 FW: gtttagtgaaccgccaccatgGGGCAGCAGCCTG;
ABL1 short REV: tcaaactgcggatggctccaAAAGGCTTGGTGGATTTCAGCAAAG;
NEF FW: gctcaagcttcgccaccatgGGGGGCAAGTGGTC;
NEF REV: tcaaactgcggatggctccaGCAGTCTTTGTAGTACTCCGGATG;
GAGpol p17 FW: gctcaagcttcgccaccatgGGTGCGAGAGCGTC;
GAGpol p17 REV: tcaaactgcggatggctccaGTAATTTTGGCTGACCTGGCTGTTG;
ARL17B FW: gctcaagcttcgccaccatgGGAAACATTTTTGAAAAGC;
ARL17B REV: tcaaactgcggatggctccaCTTTACCACAGCTGATTTC;

G2A mutants of FSP1 and DDX46 were cloned using Q5® Site-Directed Mutagenesis Kit (New England Biolabs) using the following primers:
FSP1 FW: GCCACCATGGcGTCCCAGGTC;
FSP1 REV: GAAGCTTGAGCTCGAGATCTG;
DDX46 FW: GCCACCATGGccCGGGAGTCAC;
DDX46 REV: GAAGCTTGAGCTCGAGATC.

Cell culture
HeLa and HEK293T cells were grown in low-glucose Dulbecco's Modified Eagle's Medium (DMEM) (Merck), supplemented with 10% (v/v) fetal bovine serum (FBS, Gibco). All cells were maintained in a humidified incubator at 37°C and 5% CO_2.

Metabolic labelling
HeLa or HEK293T cells were seeded into 10 cm plates or 6-well plates and grown for 24 h. Next, cells were incubated with cell growth media containing probes **YnMyr** (5 μM), **X3** (100 μM), **X8** (100 μM) or **X10** (100 μM) for additional 24 h.

UV crosslinking
After incubation, cells were carefully washed (×3) with PBS, placed on ice, and irradiated (in PBS) at 365 nm for 5 min using a Spectrolinker XL-1000 (Fisher Scientific) equipped with six 365 nm UV lamps. Irradiated cells were immediately lysed according to the protocol below.

Cell lysis
The cells were lysed with 1 mL (10 cm plates) or 200 μL (6-well plates) of lysis buffer containing 1% (v/v) Triton X-100, 0.1% (w/v) SDS, supplemented with cOmplete™ EDTA-free protease inhibitor cocktail (1×, Roche) in PBS on ice for 15 min. The lysates were clarified by centrifugation at 4°C for 10 min (at 17000 × g) and the supernatant recovered. Protein concentration was determined using the DC™ Protein Assay (Bio-Rad) in a 96-well plate as per manufacturer's instructions.

Protein denaturation
10% SDS was added to lysates to 1% final concentration. Lysates were then heated to 95˚C for 10 min.

Click chemistry (CuAAC ligation)
Click chemistry was performed as described in (12). Briefly, 0.06 volumes of click master mix was prepared by combining:

- 0.01 volume of click reagent (10 mM of AzT, AzTB or AzRB in DMSO)
- 0.02 volumes of 50 mM $CuSO_4$ in MilliQ water
- 0.02 volumes of 50 mM TCEP in MilliQ water
- 0.01 volume of 10 mM TBTA in DMSO.

0.94 volumes of lysates were diluted to 2.13 mg/mL with PBS lysis buffer (corresponding to a concentration of 2 mg/mL in the final mixture). 0.06 volumes of the click master mix were added to 0.94 volumes of protein solution and the mixture was stirred for 1 h at RT. The reaction was then quenched by addition of 0.02 volumes of 0.5 M EDTA. 2 volumes of MeOH followed by 0.5 volumes of $CHCl_3$ and followed by 1 volume of MilliQ water were then added to precipitate proteins. The precipitate was pelleted by centrifugation at 4°C for 10 min (17000 × g). The pellet was then washed twice with cold MeOH by resuspension and centrifugation. Finally, the pellet was dissolved in 0.1 volume of 2% (w/v) SDS in PBS and diluted by 0.9 volumes of PBS.

SDS-PAGE and in-gel fluorescence
Lysates (10-20 ug) were mixed with 4× Laemmli sample loading buffer (250 mM Tris-HCl pH 6.8, 30% (v/v) glycerol, 10% (w/v) SDS, 0.05% (w/v) bromophenol blue) supplemented with 20% v/v β-mercaptoethanol (BME) and incubated at 95°C for 10 min. The samples were separated on 10% (w/v) SDS-polyacrylamide gels that were cast according to manufacturer's protocol (National Diagnostics). The gels were run for 15 min at 80 V, followed by 60 min at 160 V in Tris/glycine/SDS running buffer (0.192 M glycine, 25 mM Tris base, 1% (w/v) SDS). Fluorescent images of the gels were recorded on a Typhoon™ FLA 9500 (GE Healthcare; equipped with 532 nm excitation laser and an LPG emission filter). Molecular weights were estimated using Precision Plus Protein™ All Blue Prestained Protein Standard (Bio-Rad). For protein loading quantification, after the imaging, the gels were placed in a Coomassie staining buffer (10% (w/v) ammonium sulfate, 10% (v/v) phosphoric acid, 20% (v/v) methanol, 1.2% (w/v) Coomassie brilliant blue G) overnight. The gels were then washed in MilliQ water for 1 h and images were scanned using a Canon LiDE 120 office scanner.

Western blotting
SDS-PAGE gels were prepared as described above. Proteins were transferred to nitrocellulose membranes (GE Healthcare) in transfer buffer (192 mM glycine, 25 mM Tris base, 20% (v/v) MeOH) at 100 V for 1h and transfer was checked by staining with Ponceau S. Membranes were blocked with 5% (w/v) non-fat dried skimmed milk powder in TBS-T (50 mM Tris base (pH 7.4), 150 mM NaCl, 0.1% (v/v) Tween-20) or 3% (w/v) BSA in TBS-T for 1 h at RT. Membranes were then incubated with primary antibodies in blocking buffer for 1h at RT or overnight at 4°C. Membranes were washed 3 times with TBS-T, incubated with corresponding HRP-conjugated secondary antibodies in 5 % milk or 3 % BSA in TBS-T for 1 h at RT, washed again 3 times with TBS-T and visualized using HRP substrate (Luminata Crescendo, Millipore) on an ImageQuant™ LAS-4000 Imaging System.

Immunoprecipitation-Western blotting
C-terminally TST-tagged FSP1 and DDX46 as well as the corresponding G2A mutants were transfected with Lipofectamine 2000 in HEK293T cells and overexpressed for 48 h. Cells overexpressing FSP1 were optionally treated with 1 µM **IMP-1088** (NMTi) 24 h post-transfection for additional 24 h. The cells were subsequently lysed in a buffer containing 50 mM Tris pH 8.0, 150 mM NaCl and 1% NP-40 (IGEPAL CA-630, Merck). 15 µL of Streptactin®XT magnetic beads (IBA Lifesciences) were added per 600 µg of cell lysate and incubated at 4°C for 1 h. The beads with bound proteins were washed with 100 mM Tris pH 8.0, 150 mM NaCl and proteins were eluted by boiling in 2× Laemmli buffer. Fluorescence signal from secondary antibodies (LI-COR) was quantified using the Odyssey LI-COR system. Quantification was performed using Image Studio software (LI-COR) and statistical analysis was performed using GraphPad Prism.

Streptavidin enrichment
15 µL of Dynabeads™ MyOne™ Streptavidin C1 (streptavidin beads) (Invitrogen) were washed three times with 100 µL 0.2% (w/v) SDS in PBS. Then 100 µL of 1 mg/mL "AzTB-clicked" lysates were added, and the suspension was stirred for 2 h at RT. The beads were washed three times with 1% (w/v) SDS in PBS and then resuspended in 15 µL of 1× Laemmli buffer. The tubes were placed in a heating block at 95°C for 10 min. The supernatants were then loaded onto 10% SDS-polyacrylamide gels.

Metabolic labeling and SILAC proteomics
Proteomics samples were prepared in LoBind microcentrifuge tubes (Eppendorf®). All buffer solutions were prepared fresh and filtered with a 0.22 µm syringe filter prior to use.

SILAC heavy spike-in mixture was prepared as follows. HeLa cells passaged in R10K8 SILAC heavy media (Dundee Cell Products) supplemented with 10% dialysed FBS (Merck) for at least 5 passages were treated in separate 10 cm tissue culture dishes with **YnMyr** (5 µM), **X3** (100 µM), **X8** (100 µM), **X10** (100 µM) and incubated for 24 h in a humidified incubator at 37°C and 5% CO_2. Then, cells were lysed and protein concentration measured. Equal amounts of proteins from all four conditions were mixed to create a heavy-labeled spike-in.

HeLa cells in DMEM/10% FBS (light) media were treated in triplicates with **YnMyr** (5 µM), **X3** (100 µM), **X8** (100 µM), **X10** (100 µM) for 24 h with and without the addition of 0.5 µM of **IMP-366** (DDD85646) NMT inhibitor (NMTi). Cells were lysed and protein concentration measured as described above. 250 µg of heavy spike-in was added to 250 µg of each lysate. The proteins were ligated to AzRB regent using click chemistry as described above.

30 µL of NeutrAvidin® Agarose Resin slurry (Thermo Fisher Scientific) were washed (×3) with 500 µL of 0.2% (w/v) SDS in PBS by vortexing for 1 min and centrifugation in a microcentrifuge for 1 min. 1 mL of 1 mg/mL protein solution from AzRB click chemistry reaction was added to the beads and they were stirred at for 2 h at RT. The beads were washed (×3) with 500 µL 1% (w/v) SDS in PBS, followed by (×2) with 4 M urea in 50 mM NH_4HCO_3 (AMBIC), followed by (×3) with 50 mM (AMBIC). The beads were resuspended in 50 µL of 50 mM AMBIC and 5 µL of 100 mM TCEP and were stirred for 1 h at 37°C in a thermomixer (Eppendorf®). The beads were pelleted in a microcentrifuge and the supernatant was swapped for another 50 µL of 50 mM AMBIC and 5 µL of 100 mM iodoacetamide and left in the dark without stirring for 30 min. The beads were washed (×2) with 500 µL of 50 mM AMBIC. 50 µL of 50 mM AMBIC was again added to the beads followed by 1 µL of trypsin solution (20 µg Sequencing

Grade Modified Trypsin from Promega vials were resuspended in 100 µL of 50 mM AMBIC) and left shaking in a thermomixer at 37°C overnight. The supernatant was collected, and the beads resuspended in 70 µL of 50 mM AMBIC and stirred for 15 min. The supernatant was collected and combined with a previous fraction. The beads were resuspended in 70 µL of 1.5% (v/v) TFA in MilliQ water and stirred for 15 min. The supernatant was collected again and combined with previous fractions. The peptides solution was desalted on a STAGE (stop-and-go extraction) tip. A stack of three Empore™ SDB XC polystyrene-divinylbenzene copolymer extraction disks (Merck) was cut and fit into a p200 pipette tip. The tips were activated with 150 µL of MeOH by centrifugation for 2 min at 2000 × g and washed with 150 µL of MilliQ water for 2 min at 2000 × g. The peptide samples were loaded onto the tips and centrifuged for 2 min at 2000 × g. The tips were washed with 150 µL of MilliQ water for 2 min at 2000 × g. The peptides were eluted from the tips using 60 µL of 79% MeCN in water by centrifugation for 2 min at 2000 × g. The samples were then dried in a Savant SPD1010 SpeedVac Concentrator (Thermo Scientific™) and stored at -80°C for subsequent proteomics analysis.

UV-crosslinking and SILAC proteomics

SILAC heavy spike-in mixture was prepared as follows. HeLa cells passaged in R10K8 SILAC heavy media (Dundee Cell Products) supplemented with 10% dialysed FBS (Merck) for at least 5 passages were treated in separate 10 cm tissue culture dishes with either **X3** (100 µM) or **X10** (100 µM), incubated for 24 h, UV crosslinked and lysed. HeLa cells in DMEM/10% FBS (light) media were treated in triplicates with either **X3** (100 µM) or **X10** (100 µM) for 24 h with or without the addition of 1 µM of **IMP-366** (DDD85646). Cells were UV crosslinked, lysed and protein concentration measured. 200 µg of heavy spike-in was added to 400 µg of each lysate. The proteins were ligated to AzRB regent using click chemistry as described above. Enrichment and sample preparation was performed as described above for the metabolic labeling SILAC proteomics experiment**.**

Label-free interactome proteomics

HEK293 cells were plated into 6 cm tissue culture dishes and incubated overnight. Cells were transfected in triplicates with plasmids (1-4 mg) expressing a protein of interest using Lipofectamine LTX (3-12 µL) with PLUS reagent (Invitrogen) in Opti-MEM® (Gibco) according to the manufacturer's protocol. After 6 h post-transfection, media was removed, replaced with fresh DMEM/10% FBS and cells were treated with 100 µM **X10.** After 24 h (30 h post-transfection) cells were washed (×3) with PBS, placed on ice and UV crosslinked. The cells were gently scraped, collected in a pellet and centrifuged for 3 min at 1000 × g.

60 µL of 1% (w/v) SDS in PBS was added to the pellets to initiate lysis. Lysates were then denaturated at 95˚C for 10 min to remove non-covalent interactions. After cooling to RT, 240 µL of 1.25% (v/v) Triton X-100 in PBS was added. DNA was broken down by repeatedly passing the lysates through 25G needles. The lysates were then centrifuged at 4˚C for 15 min (at 17000 × g). Supernatant was collected and protein concentration measured.

32 µL of StrepTactin beads (MagStrep "type3" XT from IBA Lifesciences) per sample were washed (×3) with 0.2% (w/v) SDS, 1% (v/v) Triton X-100 in PBS. 400 µg of lysate proteins were added and incubated with the beads at 4˚C overnight. The beads were then washed (×3) with 500 µL 0.2% (w/v) SDS, 1% (v/v) Triton X-100 in PBS, followed by (×3) with 50 mM AMBIC. The beads were resuspended in 50 µL of 50 mM AMBIC containing 10 mM TCEP and were stirred for 1 h at 37°C in a thermomixer (Eppendorf®). The beads were pelleted in a microcentrifuge and the supernatant was swapped for 50 µL of 50 mM AMBIC and 5 µL of 10 mM iodoacetamide and left in the dark without stirring for 30 min. The beads were washed (×2) with 500 µL of 50 mM AMBIC. 50 µL of 50 mM AMBIC was again added to the beads followed by trypsin solution containing 0.11 µg of trypsin (20 µg Sequencing Grade Modified Trypsin from Promega vials were resuspended in 100 µL of 50 mM AMBIC) and left shaking in a thermomixer at 37°C for 4 h. The supernatant was collected, and the beads were resuspended in 70 µL of 50 mM AMBIC and stirred for 15 min. The supernatant was collected and combined with a previous fraction. The beads were resuspended in 70 µL of 1.5% (v/v) TFA in MilliQ water and stirred for 15 minutes. The supernatant was collected again and combined with previous fractions.

The peptides solution was desalted on a STAGE (stop-and-go extraction) tip. A stack of three Empore™ SDB XC polystyrene-divinylbenzene copolymer extraction disks (Merck) was cut and fit into a p200

pipette tip. The tips were activated with 150 µL of MeOH by centrifugation for 2 min at 2000 × g and washed with 150 µL of MilliQ water for 2 min at 2000 × g. The peptide samples were loaded onto the tips and centrifuged for 2 min at 2000 × g. The tips were washed with 150 µL of MilliQ water for 2 min at 2000 × g. The peptides were eluted from the tips using 60 µL of 79% MeCN in water by centrifugation for 2 min at 2000 × g. The samples were then dried in a Savant SPD1010 SpeedVac Concentrator (Thermo Scientific™) and stored at -80°C for subsequent proteomics analysis.

Shotgun proteomics
Dried peptide samples were dissolved in 15 µL of Optima™ LC/MS H_2O (Fisher Scientific) containing 2% (v/v) UPLC grade MeCN and 0.5% (v/v) TFA, vortexed and sonicated. 3 µL of the solution were injected into the LC-MS/MS system. 3 µL of the peptides sample was injected and separated on an EASY-Spray™ Acclaim PepMap C18 column (50 cm × 75 µm inner diameter) (Thermo Fisher Scientific) using a 2-hour gradient method with solvents A (2% MeCN supplemented with 0.1% formic acid) and B (80% MeCN supplemented with 0.1% formic acid) at a flow rate of 250 nL/min. Liquid chromatography was coupled to a QExactive mass spectrometer via an easy-spray source (Thermo Fisher Scientific). The QExactive was operated in data-dependent mode with survey scans acquired at a resolution of 70,000 at m/z 200. Scans were acquired from 350 to 1800 m/z. Up to 10 of the most abundant isotope patterns with charge +2 or higher from the survey scan were selected with an isolation window of 3.0 m/z and fragmented by HCD with normalized collision energies of 25 W. The maximum ion injection times for the survey scan and the MS/MS scans (acquired with a resolution of 17,500 at m/z 200) were 250 and 80 ms, respectively; the ion target value for MS was set to 10^6 and for MS/MS to 10^5.

Proteomics data processing (MaxQuant, Perseus and PEAKS)
Proteomics results (*.RAW files) were processed using MaxQuant (version 1.5.5.1) or PEAKS 8.0 using latest releases of *Homo sapiens* proteome from uniprot.org.

In MaxQuant, digestion was set as Trypsin/P, oxidation (M) and acetylation (N-term) were set as variable modifications, carbamidomethylation (C) was set as fixed modification and other settings were as by default. For SILAC quantification in MaxQuant multiplicity was set to 2 and corresponding arginine and lysine labels were selected.

In PEAKS, digestion was set as Trypsin, precursor mass tolerance was set at 5.0 ppm, fragment ion mass tolerance was set 0.1 Da. Oxidation (M) and acetylation (N-term) were set as variable modifications, carbamidomethylation (C) was set as fixed modification. PEAKS PTM was used to find additional peptide modifications: Myr' (ΔM=463.2907, at N-terminal glycines), Myr'-N2 (ΔM=489.2812, at N-terminal glycines) and Myr-Xlink (ΔM=479.2856, at any residue).

Perseus 1.5.5.3 was used to analyze files proteinGroups.txt obtained as result of MaxQuant searches. Potential contaminants, reverse and only identified by site proteins were discarded before the analysis. All data was transformed using log2(x) function before any further manipulation. Data was normalized by subtracting a median of each data column.

t-test was performed using default Perseus settings, volcano plot representations have a visual cut-off line with parameters: s0=0.1, FDR=0.05, unless stated otherwise.

GraphPad Prism 8 was used to visualize the data.

StringDB analysis of protein interactions
Proteins significantly enriched in the DDX46-TST transfected sample (DDX46, ZC3H13, PPIG, SRRM2, TRA2A, TRA2B, ACIN1, LMAN1) were used as input to StringDB. The graph was built using default parameters and with 5 additional interactors shown.

Full gels corresponding to Figure 1C:

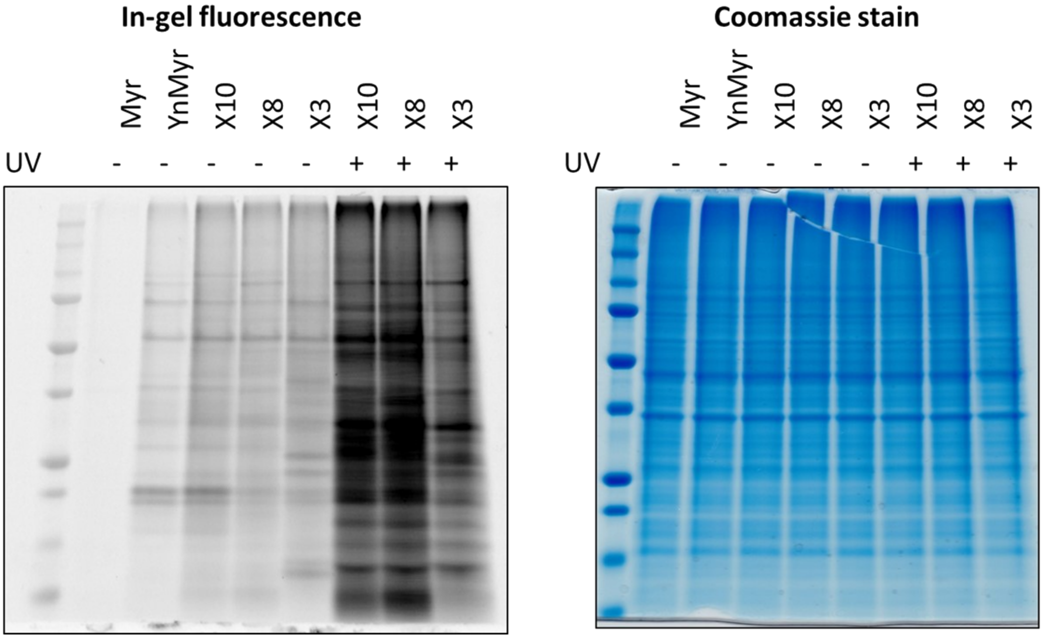

Full gels corresponding to Figure 1D:

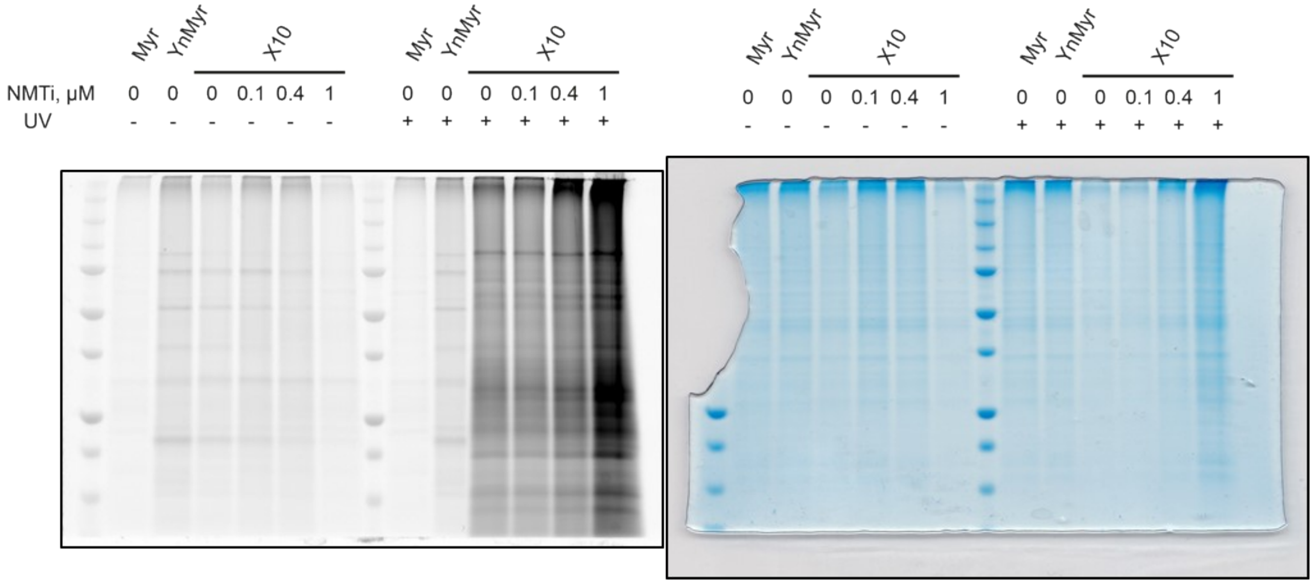

Full Western blots corresponding to Figure 1E:

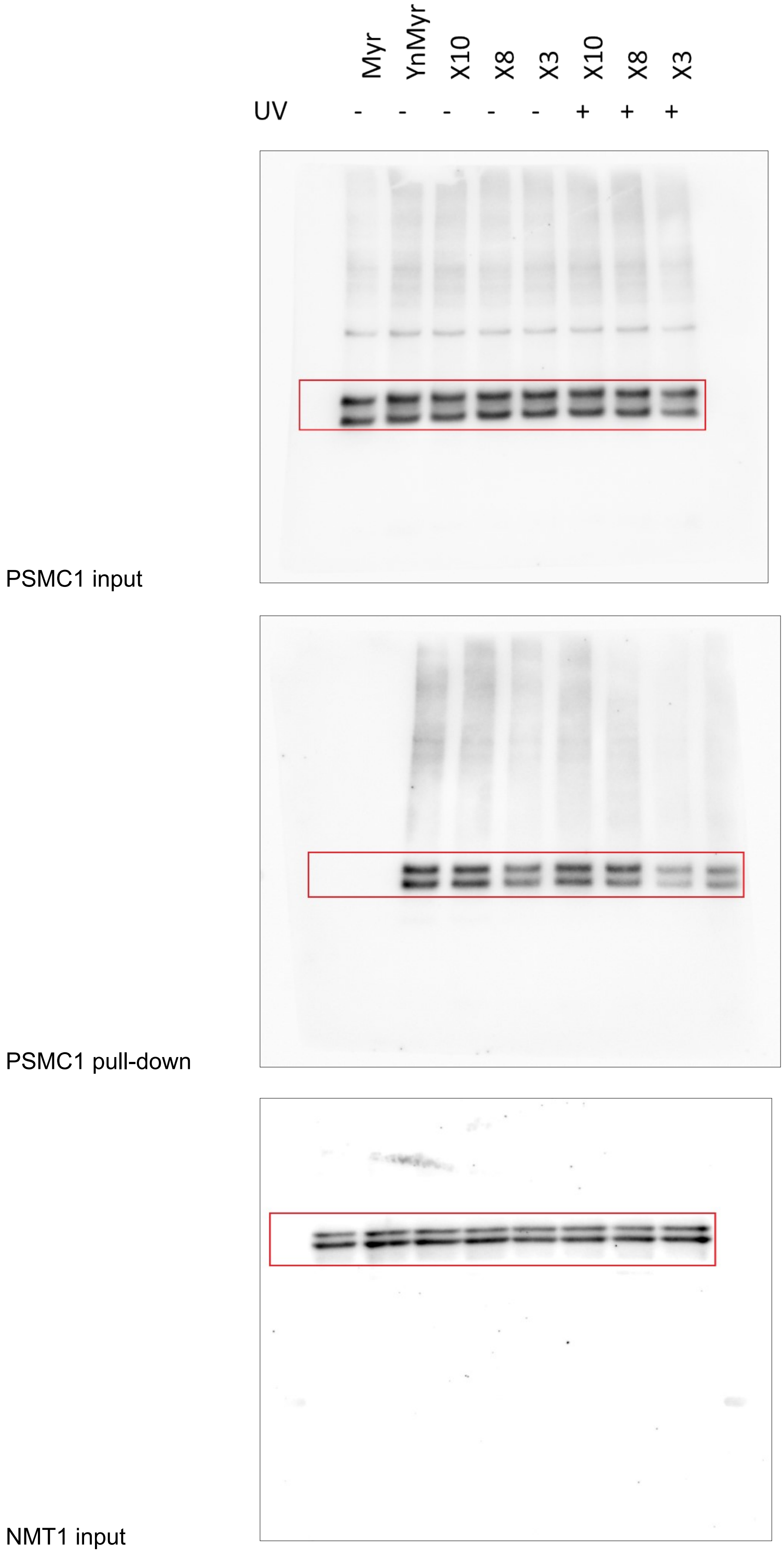

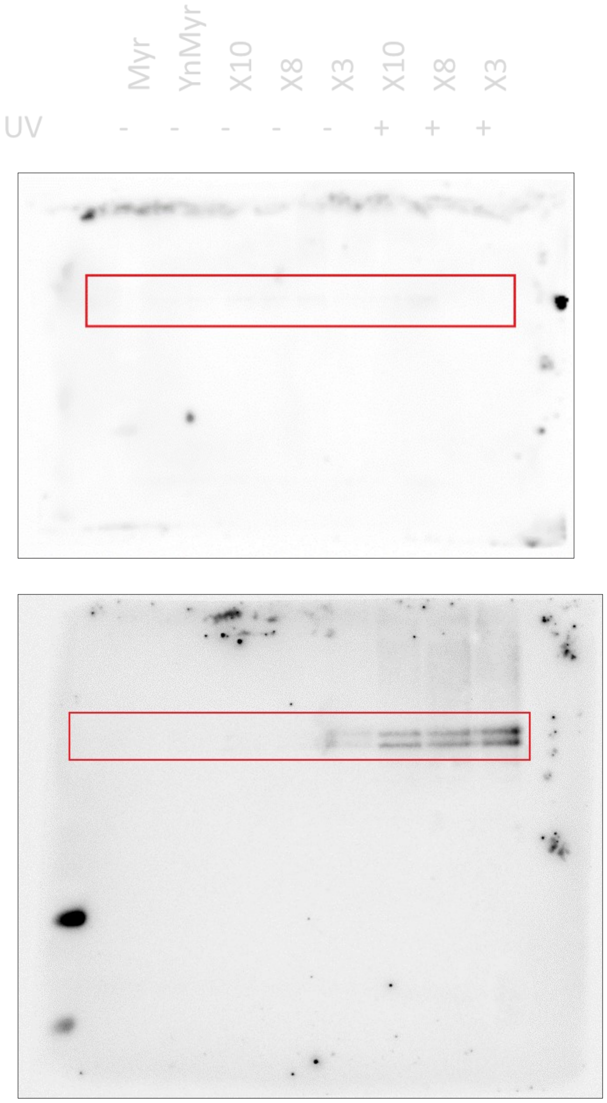

NMT1 pull-down, native

NMT1 pull-down, denatured

Full Western blots corresponding to Figure 5E

Replicate 210203 – shown on Figure 5E:

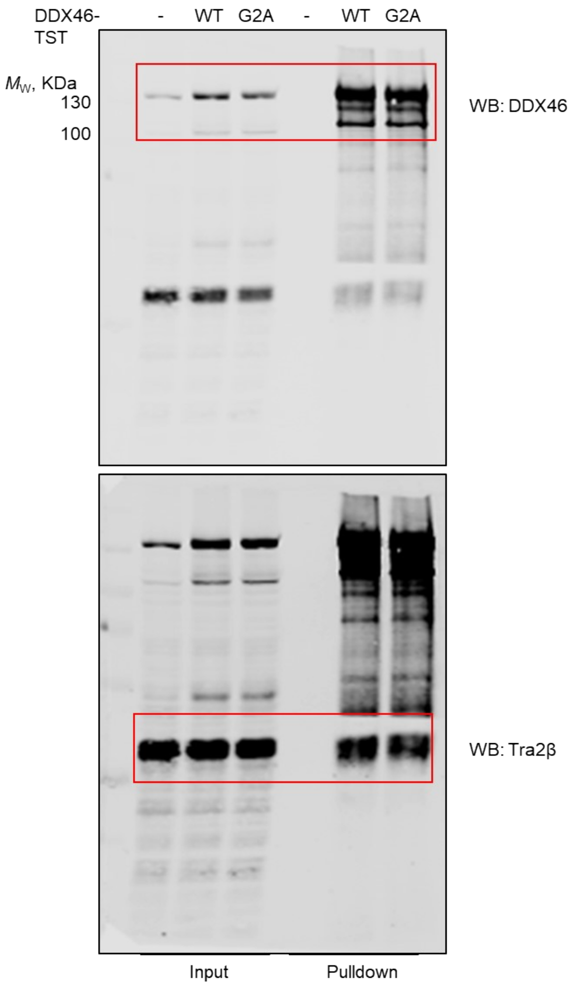

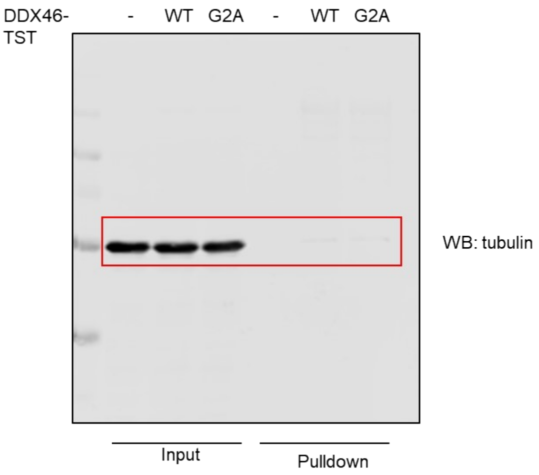

Replicate 210129:

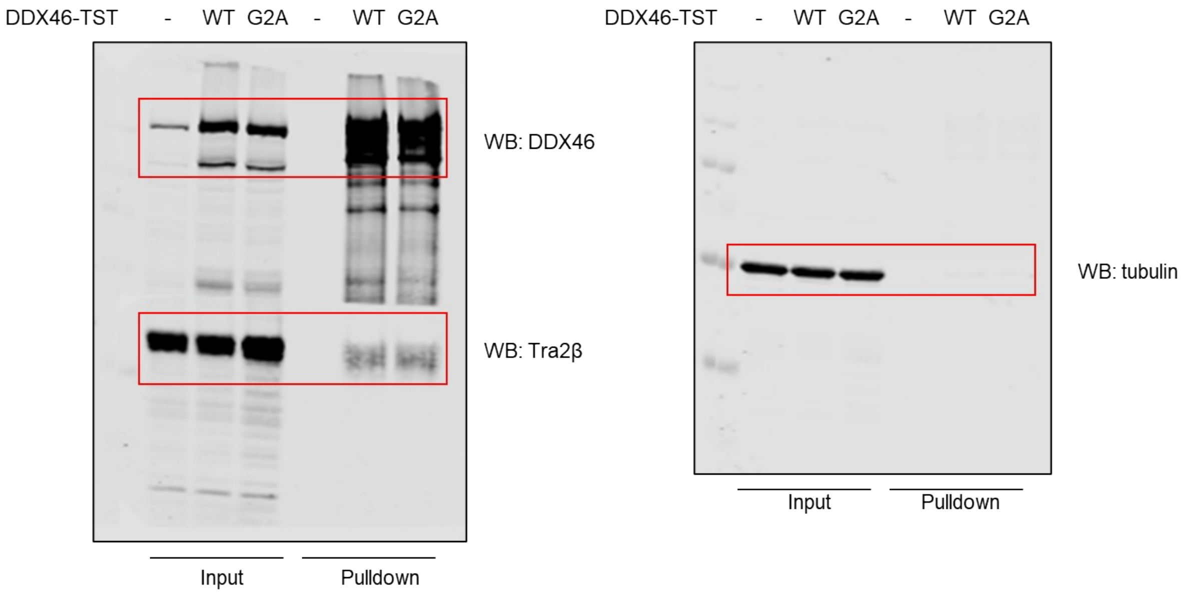

Replicate 201227:

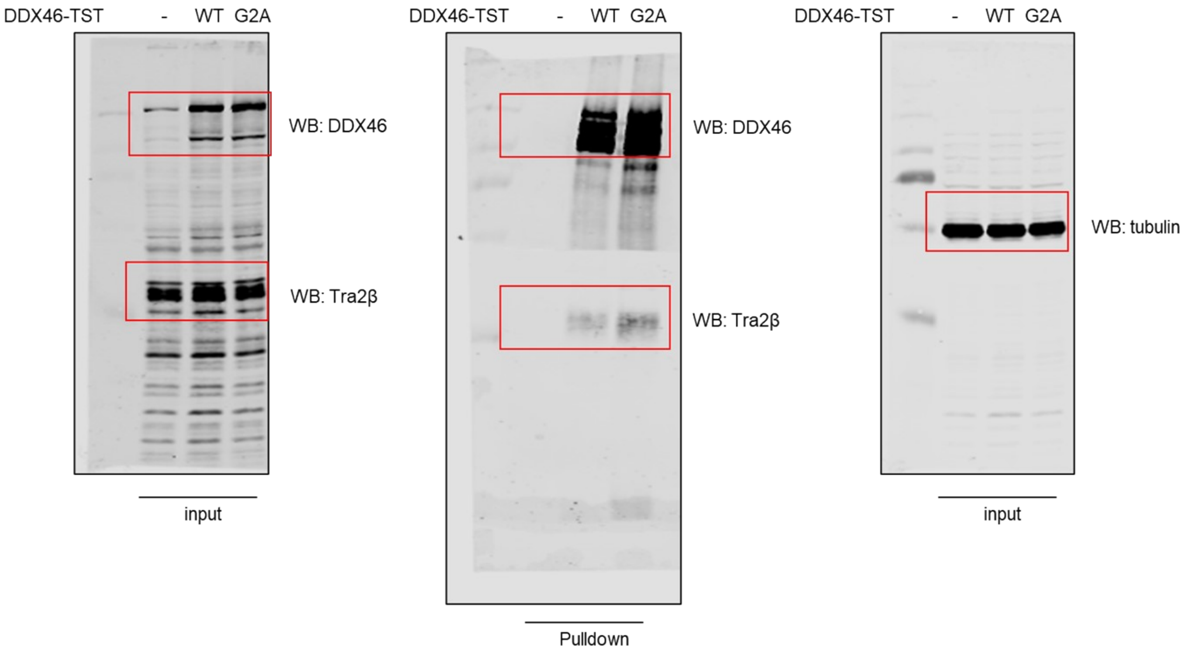

Full Western blots corresponding to Figure 5F

Replicate 210316 – shown on Figure 5F:

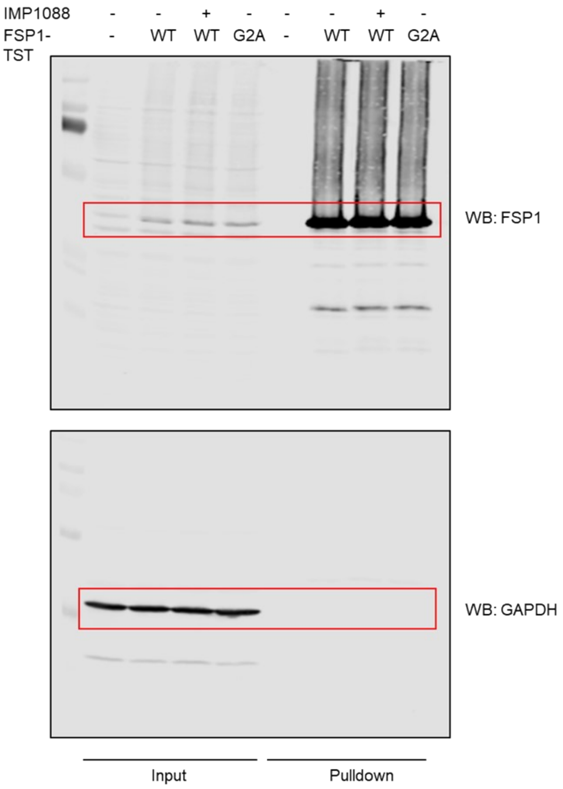

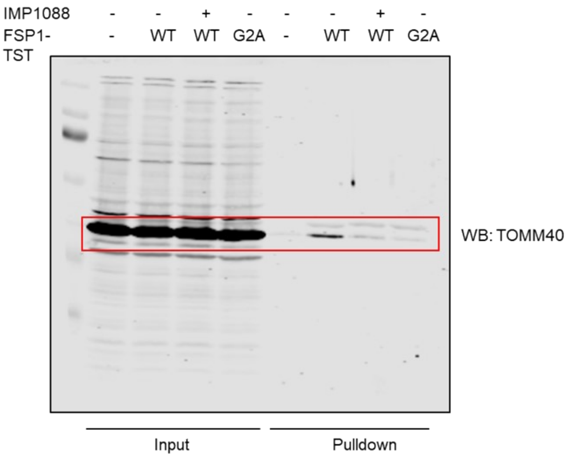

Replicate 210312:

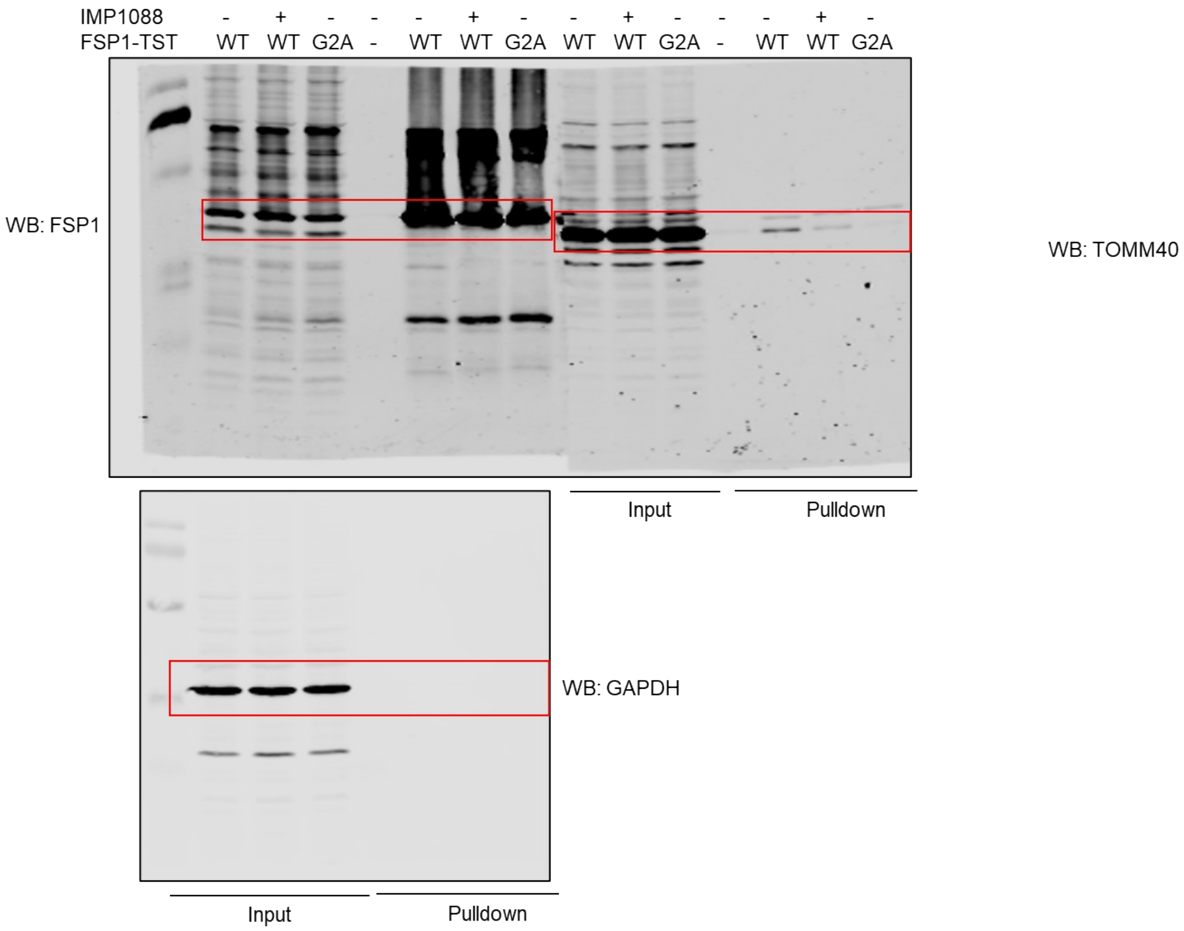

Replicates 210309-1 and 210309-2:

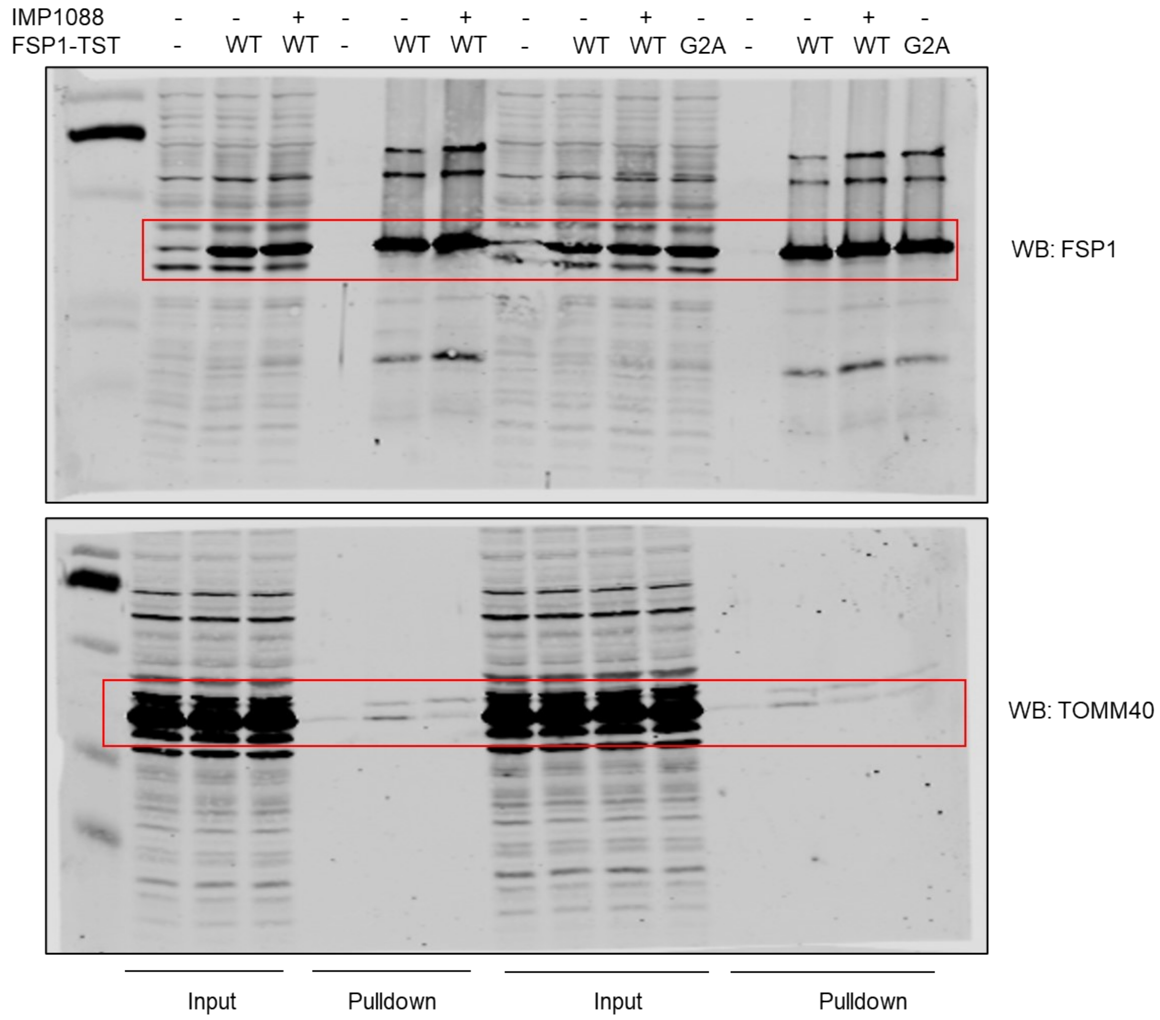

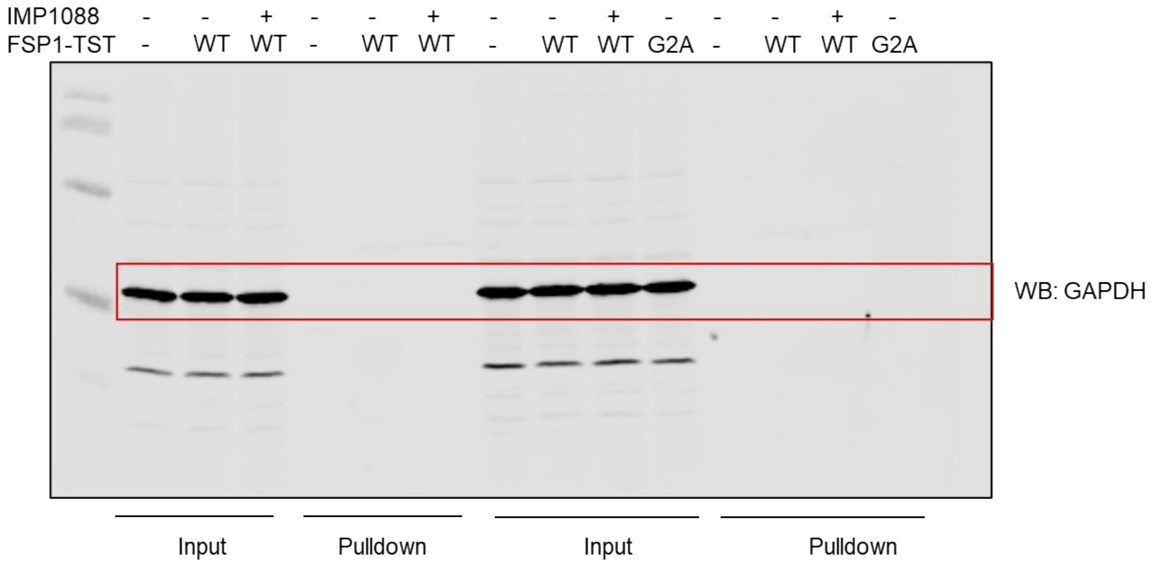

Replicate 201217:

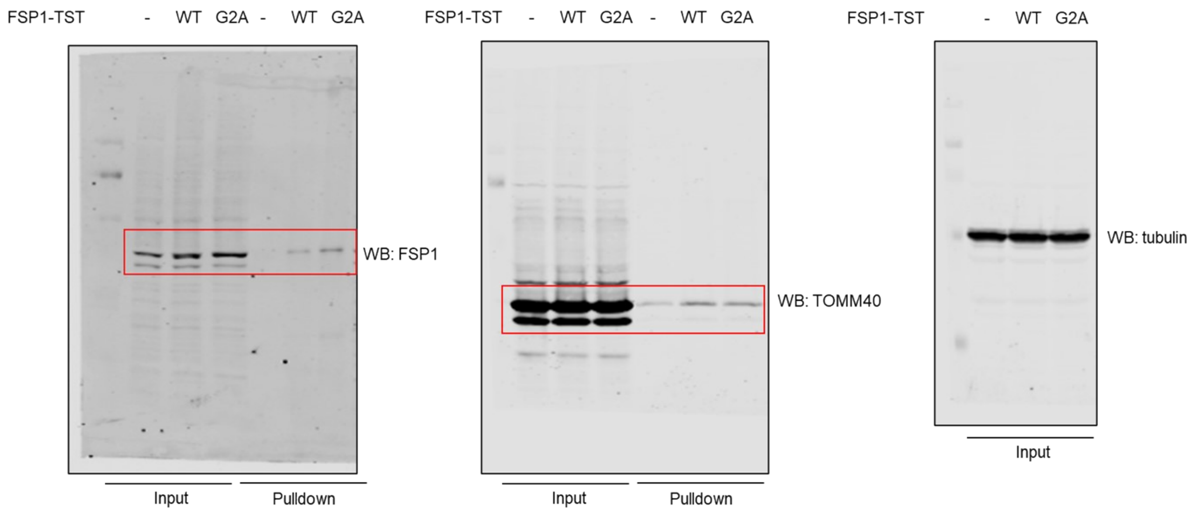

Quantification of Western blots on Figures 5E, 5F

**Replicate 201217**	TOM40	FSP1	TOM40/ FSP1	TOM40/ FSP1,%
WT	2440	62	39.35	100
G2A	2160	129	16.74	42.5
**Replicate 210316**	TOM40	FSP1	TOM40/ FSP1	TOM40/ FSP1,%
WT	1300	1E+05	0.010484	100
WT1088	253	1E+05	0.002126	20.3
G2A	115	1E+05	0.001000	9.5
**Replicate 210312**	TOM40	FSP1	TOM40/ FSP1	TOM40/ FSP1,%
WT	259	70900	0.003653	100
WT1088	69	59000	0.001169	32.0
G2A	16.2	64000	0.000253	6.9
**Replicate 210309-2**	TOM40	FSP1	TOM40/ FSP1	TOM40/ FSP1,%
WT	185	34400	0.005378	100
WT1088	56.3	36300	0.001551	28.8
**Replicate 210309-1**	TOM40	FSP1	TOM40/ FSP1	TOM40/ FSP1,%
WT	122	34000	0.003588	100
WT1088	51.5	45000	0.001144	31.9
G2A	33	31600	0.001044	29.1

**Replicate 201217**	DDX46	Tra2b	Tra2b/ DDX46	Tra2b/ DDX46,%
WT	43600	1220	0.0280	100
G2A	61100	2830	0.0463	165.5
**Replicate 210129**	DDX46	Tra2b	Tra2b/ DDX46	Tra2b/ DDX46,%
WT	88000	8320	0.0945	100
G2A	84800	7960	0.0939	99.3
**Replicate 210203**	DDX46	Tra2b	Tra2b/ DDX46	Tra2b/ DDX46,%
WT	1E+05	7330	0.0573	100
G2A	1E+05	6610	0.0590	103.1

References

1. Szklarczyk D, Gable AL, Lyon D, Junge A, Wyder S, Huerta-Cepas J, et al. STRING v11: protein–protein association networks with increased coverage, supporting functional discovery in genome-wide experimental datasets. Nucleic Acids Res. 2019 Jan;47(D1):D607–13.

2. Baggelaar MP, Janssen FJ, van Esbroeck ACM, den Dulk H, Allarà M, Hoogendoorn S, et al. Development of an Activity-Based Probe and In Silico Design Reveal Highly Selective Inhibitors for Diacylglycerol Lipase-α in Brain. Angew Chemie Int Ed. 2013 Nov;52(46):12081–5.

3. Wirtz L, Auerbach D, Jung G, Kazmaier U. Fluorescence Labeling of Amino Acids and Peptides with 7-Aminocoumarins. Synthesis (Stuttg). 2012 Jul;44(13):2005–12.

4. Li Z, Hao P, Li L, Tan CYJ, Cheng X, Chen GYJ, et al. Design and Synthesis of Minimalist Terminal Alkyne-Containing Diazirine Photo-Crosslinkers and Their Incorporation into Kinase Inhibitors for Cell- and Tissue-Based Proteome Profiling. Angew Chemie. 2013;125(33):8713–8.

5. Abo M, Weerapana E. A Caged Electrophilic Probe for Global Analysis of Cysteine Reactivity in Living Cells. J Am Chem Soc. 2015 Jun;137(22):7087–90.

6. Tamaru Y, Ochiai H, Sanda F, Yoshida Z. A convenient and efficient unsymmetrical ketone synthesis from acid chlorides and alkyl iodides catalyzed by palladium. Tetrahedron Lett. 1985 Jan;26(45):5529–32.

7. Cotton A, Smith D. (Triphenylphosphine)palladium (0). In: Inorganic syntheses. 1972. p. 121–4.

8. Heal WP, Wickramasinghe SR, Leatherbarrow RJ, Tate EW. N-Myristoyl transferase-mediated protein labelling in vivo. Org Biomol Chem. 2008;6(13):2308.

9. Dian C, Pérez-Dorado I, Rivière F, Asensio T, Legrand P, Ritzefeld M, et al. High-resolution snapshots of human N-myristoyltransferase in action illuminate a mechanism promoting N-terminal Lys and Gly myristoylation. Nat Commun. 2020;11(1):1–15.

10. Goncalves V, Brannigan J a, Thinon E, Olaleye TO, Serwa R, Lanzarone S, et al. A fluorescence-based assay for N-myristoyltransferase activity. Anal Biochem. 2012 Feb;421(1):342–4.

11. Broncel M, Serwa RA, Ciepla P, Krause E, Dallman MJ, Magee AI, et al. Multifunctional reagents for quantitative proteome-wide analysis of protein modification in human cells and dynamic profiling of protein lipidation during vertebrate development. Angew Chemie - Int Ed. 2015;54(20):5948–51.

12. Thinon E, Serwa RA, Broncel M, Brannigan JA, Brassat U, Wright MH, et al. Global profiling of co- and post-translationally N-myristoylated proteomes in human cells. Nat Commun. 2014 Dec;5(1):4919.

13. Kabsch W. Automatic processing of rotation diffraction data from crystals of initially unknown symmetry land cell constants. J Appl Crystallogr. 1993;26(pt 6):795–800.

14. Evans PR. An introduction to data reduction: space-group determination, scaling and intensity statistics. Acta Crystallogr Sect D Biol Crystallogr. 2011 Apr;67(4):282–92.

15. McCoy AJ, Grosse-Kunstleve RW, Adams PD, Winn MD, Storoni LC, Read RJ. Phaser crystallographic software. J Appl Crystallogr. 2007 Aug;40(4):658–74.

16. Emsley P, Lohkamp B, Scott WG, Cowtan K. Features and development of Coot. Acta Crystallogr Sect D Biol Crystallogr. 2010;66(4):486–501.

17. Murshudov GN, Skubák P, Lebedev AA, Pannu NS, Steiner RA, Nicholls RA, et al. REFMAC 5 for the refinement of macromolecular crystal structures. Acta Crystallogr Sect D Biol Crystallogr. 2011 Apr;67(4):355–67.

18. Adams PD, Afonine P V., Bunkóczi G, Chen VB, Davis IW, Echols N, et al. PHENIX: A comprehensive Python-based system for macromolecular structure solution. Acta Crystallogr Sect D Biol Crystallogr. 2010;66(2):213–21.

19. Moriarty NW, Grosse-Kunstleve RW, Adams PD. electronic Ligand Builder and Optimization Workbench (eLBOW): a tool for ligand coordinate and restraint generation. Acta Crystallogr Sect D Biol Crystallogr. 2009 Oct 1;65(10):1074–80.

20. Chen VB, Arendall WB, Headd JJ, Keedy DA, Immormino RM, Kapral GJ, et al. MolProbity: All-atom structure validation for macromolecular crystallography. Acta Crystallogr Sect D Biol Crystallogr. 2010;66(1):12–21.
