# Supplementary material for: Discovery of lipid-mediated protein–protein interactions in living cells using metabolic labeling with photoactivatable clickable probes
Source: Chem Sci. 2023 Jan 30;14(9):2419–30. doi: 10.1039/d2sc06116c (PMC9977449; doi:10.1039/d2sc06116c)
Supplement: SC-014-D2SC06116C-s006 [file SC-014-D2SC06116C-s006.pdf]

Electronic Supplementary Material (ESI) for Chemical Science.
This journal is © The Royal Society of Chemistry 2023

Full wwPDB X-ray Structure Validation Report (i)

May 25, 2020 – 07:21 pm BST

PDB ID	:	5NPQ
Title	:	Human N-myristoyltransferase 1 (MNT1) with Myristoyl-CoA analogue X10 bound
Authors	:	Shen, M.; Perez-Dorado, I.; Fedoryshchak, R.; Tate, E.W.
Deposited on	:	2017-04-18
Resolution	:	2.37 Å(reported)

This is a Full wwPDB X-ray Structure Validation Report for a publicly released PDB entry.

We welcome your comments at *validation@mail.wwpdb.org*
A user guide is available at
https://www.wwpdb.org/validation/2017/XrayValidationReportHelp
with specific help available everywhere you see the (i) symbol.

The following versions of software and data (see references (i)) were used in the production of this report:

MolProbity	:	4.02b-467
Mogul	:	1.8.5 (274361), CSD as541be (2020)
Xtriage (Phenix)	:	1.13
EDS	:	2.11
buster-report	:	1.1.7 (2018)
Percentile statistics	:	20191225.v01 (using entries in the PDB archive December 25th 2019)
Refmac	:	5.8.0158
CCP4	:	7.0.044 (Gargrove)
Ideal geometry (proteins)	:	Engh & Huber (2001)
Ideal geometry (DNA, RNA)	:	Parkinson et al. (1996)
Validation Pipeline (wwPDB-VP)	:	2.11

1 Overall quality at a glance (i)

The following experimental techniques were used to determine the structure:
X-RAY DIFFRACTION

The reported resolution of this entry is 2.37 Å.

Percentile scores (ranging between 0-100) for global validation metrics of the entry are shown in the following graphic. The table shows the number of entries on which the scores are based.

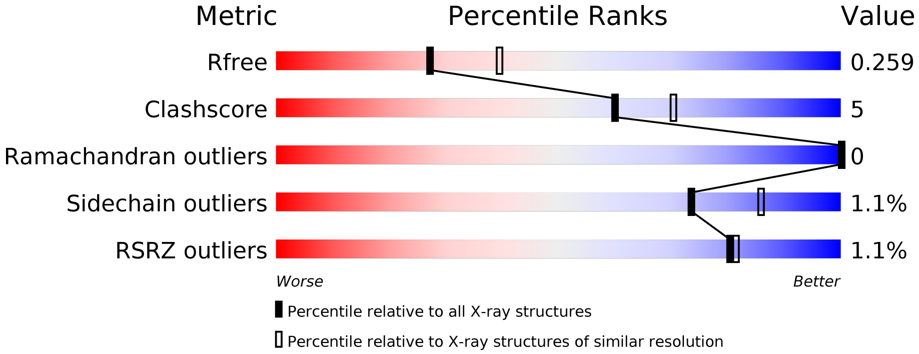

Metric	Whole archive (#Entries)	Similar resolution (#Entries, resolution range(Å))
R_{free}	130704	5509 (2.40-2.36)
Clashscore	141614	6082 (2.40-2.36)
Ramachandran outliers	138981	5973 (2.40-2.36)
Sidechain outliers	138945	5975 (2.40-2.36)
RSRZ outliers	127900	5397 (2.40-2.36)

The table below summarises the geometric issues observed across the polymeric chains and their fit to the electron density. The red, orange, yellow and green segments on the lower bar indicate the fraction of residues that contain outliers for >=3, 2, 1 and 0 types of geometric quality criteria respectively. A grey segment represents the fraction of residues that are not modelled. The numeric value for each fraction is indicated below the corresponding segment, with a dot representing fractions <=5% The upper red bar (where present) indicates the fraction of residues that have poor fit to the electron density. The numeric value is given above the bar.

Mol	Chain	Length	Quality of chain
1	A	391	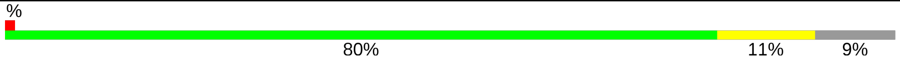
1	B	391	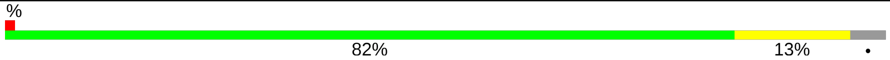

2 Entry composition

There are 5 unique types of molecules in this entry. The entry contains 6174 atoms, of which 0 are hydrogens and 0 are deuteriums.

In the tables below, the ZeroOcc column contains the number of atoms modelled with zero occupancy, the AltConf column contains the number of residues with at least one atom in alternate conformation and the Trace column contains the number of residues modelled with at most 2 atoms.

- Molecule 1 is a protein called Glycylpeptide N-tetradecanoyltransferase 1.

Mol	Chain	Residues	Atoms					ZeroOcc	AltConf	Trace
			Total	C	N	O	S			
1	A	355	2843	1850	477	505	11	0	0	0
1	B	375	3046	1978	510	544	14	0	0	0

There are 6 discrepancies between the modelled and reference sequences:

Chain	Residue	Modelled	Actual	Comment	Reference
A	106	GLY	-	expression tag	UNP P30419
A	107	PRO	-	expression tag	UNP P30419
A	108	HIS	-	expression tag	UNP P30419
B	106	GLY	-	expression tag	UNP P30419
B	107	PRO	-	expression tag	UNP P30419
B	108	HIS	-	expression tag	UNP P30419

- Molecule 2 is GLYCEROL (three-letter code: GOL) (formula: $C_3H_8O_3$).

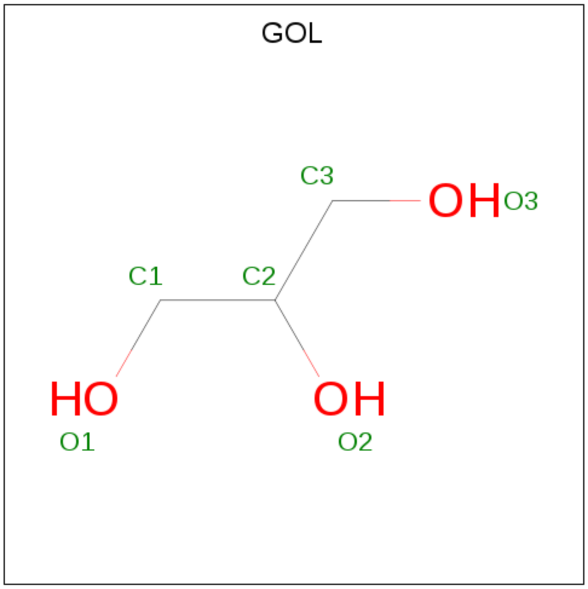

Mol	Chain	Residues	Atoms	ZeroOcc	AltConf
2	A	1	Total C O<br>6 3 3	0	0
2	A	1	Total C O<br>6 3 3	0	0
2	B	1	Total C O<br>6 3 3	0	0
2	B	1	Total C O<br>6 3 3	0	0

- Molecule 3 is {S}-[2-[3-[[(2 {S})-4-[[[(2 {R},3 {S},4 {R},5 {R})-5-(6-aminopurin-9-yl)-4-oxidanyl-3-phosphonooxy-oxolan-2-yl]methoxy-oxidanyl-phosphoryl]oxy-oxidanyl-phosphoryl]oxy-3,3-dimethyl-2-oxidanyl-butanoyl]amino]propanoylamino]ethyl] 9-(3-but-3-ynyl-1,2-diazirin-3-yl)nonanethioate (three-letter code: 94Q) (formula: $C_{35}H_{56}N_{9}O_{17}P_{3}S$).

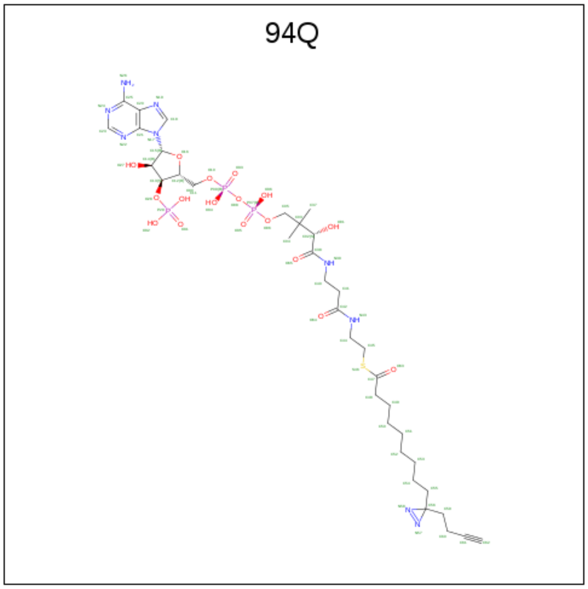

Mol	Chain	Residues	Atoms	ZeroOcc	AltConf
3	A	1	Total C N O P S<br>65 35 9 17 3 1	0	0
3	B	1	Total C N O P S<br>65 35 9 17 3 1	0	0

- Molecule 4 is MAGNESIUM ION (three-letter code: MG) (formula: Mg).

Mol	Chain	Residues	Atoms	ZeroOcc	AltConf
4	B	1	Total Mg<br>1 1	0	0
4	A	1	Total Mg<br>1 1	0	0

- Molecule 5 is water.

Mol	Chain	Residues	Atoms	ZeroOcc	AltConf
5	A	77	Total O<br>77 77	0	0
5	B	52	Total O<br>52 52	0	0

3 Residue-property plots (i)

These plots are drawn for all protein, RNA and DNA chains in the entry. The first graphic for a chain summarises the proportions of the various outlier classes displayed in the second graphic. The second graphic shows the sequence view annotated by issues in geometry and electron density. Residues are color-coded according to the number of geometric quality criteria for which they contain at least one outlier: green = 0, yellow = 1, orange = 2 and red = 3 or more. A red dot above a residue indicates a poor fit to the electron density (RSRZ > 2). Stretches of 2 or more consecutive residues without any outlier are shown as a green connector. Residues present in the sample, but not in the model, are shown in grey.

• Molecule 1: Glycylpeptide N-tetradecanoyltransferase 1

Chain A:

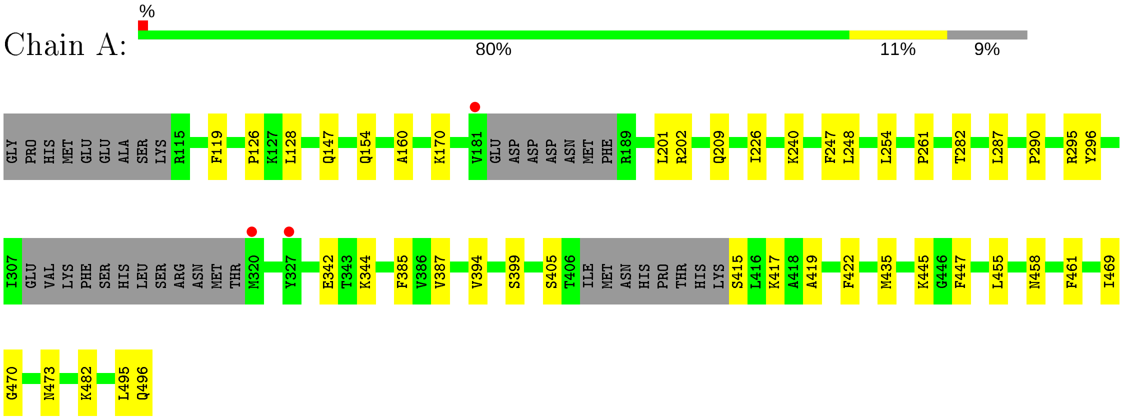

• Molecule 1: Glycylpeptide N-tetradecanoyltransferase 1

Chain B:

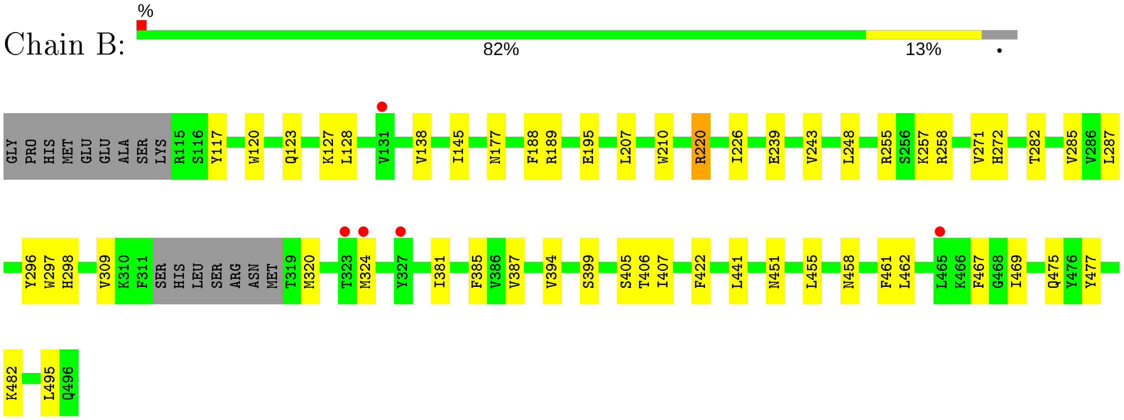

4 Data and refinement statistics (i)

Property	Value	Source
Space group	P 21 21 2	Depositor
Cell constants<br>a, b, c, α, β, γ	79.97Å 177.45Å 58.24Å<br>90.00° 90.00° 90.00°	Depositor
Resolution (Å)	88.73 – 2.37<br>88.73 – 2.37	Depositor<br>EDS
% Data completeness<br>(in resolution range)	100.0 (88.73-2.37)<br>100.0 (88.73-2.37)	Depositor<br>EDS
R_{merge}	0.19	Depositor
R_{sym}	(Not available)	Depositor
$< I/\sigma(I) >$ [1]	1.96 (at 2.37Å)	Xtriage
Refinement program	PHENIX 1.10.1_2155	Depositor
R, R_{free}	0.199 , 0.261<br>0.200 , 0.259	Depositor<br>DCC
R_{free} test set	1735 reflections (5.04%)	wwPDB-VP
Wilson B-factor (Å^2)	32.7	Xtriage
Anisotropy	0.347	Xtriage
Bulk solvent k_{sol}(e/Å^3), B_{sol}(Å^2)	0.38 , 48.3	EDS
L-test for twinning[2]	$< \|L\| > = 0.45$, $< L^2 > = 0.27$	Xtriage
Estimated twinning fraction	No twinning to report.	Xtriage
F_o,F_c correlation	0.94	EDS
Total number of atoms	6174	wwPDB-VP
Average B, all atoms (Å^2)	34.0	wwPDB-VP

Xtriage's analysis on translational NCS is as follows: *The largest off-origin peak in the Patterson function is 15.32% of the height of the origin peak. No significant pseudotranslation is detected.*

[1] Intensities estimated from amplitudes.

[2] Theoretical values of $< |L| >$, $< L^2 >$ for acentric reflections are 0.5, 0.333 respectively for untwinned datasets, and 0.375, 0.2 for perfectly twinned datasets.

5 Model quality (i)

5.1 Standard geometry (i)

Bond lengths and bond angles in the following residue types are not validated in this section: GOL, MG, 94Q

The Z score for a bond length (or angle) is the number of standard deviations the observed value is removed from the expected value. A bond length (or angle) with $|Z| > 5$ is considered an outlier worth inspection. RMSZ is the root-mean-square of all Z scores of the bond lengths (or angles).

Mol	Chain	Bond lengths		Bond angles	
		RMSZ	#|Z| >5	RMSZ	#|Z| >5
1	A	0.47	1/2923 (0.0%)	0.59	0/3980
1	B	0.43	1/3133 (0.0%)	0.57	0/4263
All	All	0.45	2/6056 (0.0%)	0.58	0/8243

All (2) bond length outliers are listed below:

Mol	Chain	Res	Type	Atoms	Z	Observed(Å)	Ideal(Å)
1	A	147	GLN	C-N	-7.27	1.17	1.34
1	B	123	GLN	C-N	5.21	1.44	1.34

There are no bond angle outliers.

There are no chirality outliers.

There are no planarity outliers.

5.2 Too-close contacts (i)

In the following table, the Non-H and H(model) columns list the number of non-hydrogen atoms and hydrogen atoms in the chain respectively. The H(added) column lists the number of hydrogen atoms added and optimized by MolProbity. The Clashes column lists the number of clashes within the asymmetric unit, whereas Symm-Clashes lists symmetry related clashes.

Mol	Chain	Non-H	H(model)	H(added)	Clashes	Symm-Clashes
1	A	2843	0	2780	26	0
1	B	3046	0	3006	29	0
2	A	12	0	16	0	0
2	B	12	0	16	0	0
3	A	65	0	0	0	0
3	B	65	0	0	0	0
4	A	1	0	0	0	0

Continued on next page...

Continued from previous page...

Mol	Chain	Non-H	H(model)	H(added)	Clashes	Symm-Clashes
4	B	1	0	0	0	0
5	A	77	0	0	2	0
5	B	52	0	0	1	0
All	All	6174	0	5818	55	0

The all-atom clashscore is defined as the number of clashes found per 1000 atoms (including hydrogen atoms). The all-atom clashscore for this structure is 5.

All (55) close contacts within the same asymmetric unit are listed below, sorted by their clash magnitude.

Atom-1	Atom-2	Interatomic distance (Å)	Clash overlap (Å)
1:B:145:ILE:HD12	1:B:272:HIS:HB3	1.67	0.75
1:B:458:ASN:HA	1:B:461:PHE:CE2	2.39	0.57
1:A:295:ARG:NH1	1:A:470:GLY:O	2.40	0.55
1:B:298:HIS:ND1	1:B:451:ASN:OD1	2.39	0.55
1:B:399:SER:OG	1:B:422:PHE:HB3	2.07	0.54
1:B:138:VAL:HG22	1:B:482:LYS:HG3	1.88	0.54
1:A:342:GLU:OE1	1:A:344:LYS:HE2	2.08	0.54
1:B:285:VAL:HG23	1:B:287:LEU:HD21	1.91	0.53
1:B:226:ILE:HD12	1:B:248:LEU:HD13	1.91	0.52
1:A:405:SER:O	1:A:415:SER:OG	2.27	0.51
1:B:188:PHE:HA	1:B:406:THR:O	2.09	0.51
1:A:240:LYS:HD3	5:A:626:HOH:O	2.10	0.51
1:B:239:GLU:OE1	5:B:601:HOH:O	2.20	0.49
1:B:458:ASN:O	1:B:462:LEU:HG	2.12	0.48
1:B:243:VAL:HG21	1:B:271:VAL:HG21	1.95	0.47
1:B:220:ARG:HA	1:B:220:ARG:HD3	1.68	0.47
1:B:195:GLU:HB3	1:B:381:ILE:HD11	1.97	0.46
1:B:177:ASN:O	1:B:189:ARG:HD2	2.14	0.46
1:B:117:TYR:HB3	1:B:120:TRP:HB2	1.98	0.46
1:B:475:GLN:HB2	1:B:477:TYR:CE2	2.52	0.45
1:A:202:ARG:NH2	5:A:602:HOH:O	2.27	0.45
1:A:126:PRO:HG3	1:A:482:LYS:HG2	1.98	0.45
1:B:320:MET:O	1:B:324:MET:HG3	2.17	0.44
1:B:282:THR:HG23	1:B:495:LEU:HD12	1.97	0.44
1:A:295:ARG:HG2	1:A:473:ASN:OD1	2.17	0.44
1:A:387:VAL:O	1:A:394:VAL:HA	2.18	0.44
1:A:119:PHE:CD1	1:A:261:PRO:HB3	2.53	0.44
1:A:128:LEU:HA	1:A:128:LEU:HD23	1.85	0.43
1:A:201:LEU:HD21	1:A:247:PHE:CD2	2.54	0.43
1:A:458:ASN:HA	1:A:461:PHE:CE2	2.53	0.43

Continued on next page...

Continued from previous page...

Atom-1	Atom-2	Interatomic distance (Å)	Clash overlap (Å)
1:A:419:ALA:HB2	1:A:447:PHE:CD2	2.54	0.43
1:B:128:LEU:HA	1:B:128:LEU:HD12	1.86	0.42
1:B:324:MET:HE2	1:B:324:MET:HB2	1.81	0.42
1:B:309:VAL:HG11	1:B:407:ILE:HD13	2.01	0.42
1:A:160:ALA:HB1	1:A:209:GLN:HA	2.01	0.42
1:A:435:MET:HG3	1:A:461:PHE:CZ	2.55	0.42
1:A:282:THR:HG23	1:A:495:LEU:HD12	2.01	0.42
1:A:385:PHE:HZ	1:A:445:LYS:HE3	1.84	0.42
1:B:255:ARG:O	1:B:257:LYS:HG3	2.20	0.42
1:B:385:PHE:CE1	1:B:441:LEU:HD13	2.55	0.42
1:A:295:ARG:HH11	1:A:469:ILE:HG12	1.83	0.42
1:B:207:LEU:HD12	1:B:210:TRP:CE2	2.55	0.42
1:A:287:LEU:O	1:A:290:PRO:HD3	2.20	0.41
1:B:127:LYS:HD3	1:B:127:LYS:HA	1.96	0.41
1:A:399:SER:OG	1:A:422:PHE:HB3	2.21	0.41
1:A:496:GLN:H	1:A:496:GLN:CD	2.24	0.41
1:A:201:LEU:HD21	1:A:247:PHE:HD2	1.86	0.41
1:A:417:LYS:HE2	1:A:447:PHE:CZ	2.56	0.41
1:B:462:LEU:O	1:B:467:PHE:HB2	2.21	0.41
1:A:226:ILE:HD12	1:A:248:LEU:HD13	2.03	0.41
1:B:258:ARG:HD3	1:B:258:ARG:HA	1.91	0.41
1:A:170:LYS:HE2	1:A:170:LYS:HB3	1.87	0.40
1:A:254:LEU:HD12	1:A:254:LEU:HA	1.83	0.40
1:B:297:TRP:CZ2	1:B:469:ILE:HD12	2.57	0.40
1:B:387:VAL:O	1:B:394:VAL:HA	2.22	0.40

There are no symmetry-related clashes.

5.3 Torsion angles ⓘ

5.3.1 Protein backbone ⓘ

In the following table, the Percentiles column shows the percent Ramachandran outliers of the chain as a percentile score with respect to all X-ray entries followed by that with respect to entries of similar resolution.

The Analysed column shows the number of residues for which the backbone conformation was analysed, and the total number of residues.

Mol	Chain	Analysed	Favoured	Allowed	Outliers	Percentiles	
1	A	347/391 (89%)	340 (98%)	7 (2%)	0	100	100

Continued on next page...

Continued from previous page...

Mol	Chain	Analysed	Favoured	Allowed	Outliers	Percentiles	
1	B	371/391 (95%)	364 (98%)	7 (2%)	0	100	100
All	All	718/782 (92%)	704 (98%)	14 (2%)	0	100	100

There are no Ramachandran outliers to report.

5.3.2 Protein sidechains (i)

In the following table, the Percentiles column shows the percent sidechain outliers of the chain as a percentile score with respect to all X-ray entries followed by that with respect to entries of similar resolution.

The Analysed column shows the number of residues for which the sidechain conformation was analysed, and the total number of residues.

Mol	Chain	Analysed	Rotameric	Outliers	Percentiles	
1	A	304/355 (86%)	301 (99%)	3 (1%)	76	87
1	B	333/355 (94%)	329 (99%)	4 (1%)	71	84
All	All	637/710 (90%)	630 (99%)	7 (1%)	73	86

All (7) residues with a non-rotameric sidechain are listed below:

Mol	Chain	Res	Type
1	A	154	GLN
1	A	296	TYR
1	A	455	LEU
1	B	220	ARG
1	B	296	TYR
1	B	405	SER
1	B	455	LEU

Some sidechains can be flipped to improve hydrogen bonding and reduce clashes. There are no such sidechains identified.

5.3.3 RNA (i)

There are no RNA molecules in this entry.

5.4 Non-standard residues in protein, DNA, RNA chains (i)

There are no non-standard protein/DNA/RNA residues in this entry.

5.5 Carbohydrates (i)

There are no carbohydrates in this entry.

5.6 Ligand geometry (i)

Of 8 ligands modelled in this entry, 2 are monoatomic - leaving 6 for Mogul analysis.

In the following table, the Counts columns list the number of bonds (or angles) for which Mogul statistics could be retrieved, the number of bonds (or angles) that are observed in the model and the number of bonds (or angles) that are defined in the Chemical Component Dictionary. The Link column lists molecule types, if any, to which the group is linked. The Z score for a bond length (or angle) is the number of standard deviations the observed value is removed from the expected value. A bond length (or angle) with $|Z| > 2$ is considered an outlier worth inspection. RMSZ is the root-mean-square of all Z scores of the bond lengths (or angles).

Mol	Type	Chain	Res	Link	Bond lengths			Bond angles		
					Counts	RMSZ	$\#\|Z\| > 2$	Counts	RMSZ	$\#\|Z\| > 2$
2	GOL	B	502	-	5,5,5	0.26	0	5,5,5	0.53	0
3	94Q	B	503	4	55,68,68	1.26	4 (7%)	64,98,98	1.37	7 (10%)
2	GOL	A	502	-	5,5,5	0.35	0	5,5,5	0.46	0
3	94Q	A	503	4	55,68,68	1.33	6 (10%)	64,98,98	1.99	16 (25%)
2	GOL	A	501	-	5,5,5	0.39	0	5,5,5	0.40	0
2	GOL	B	501	-	5,5,5	0.36	0	5,5,5	0.31	0

In the following table, the Chirals column lists the number of chiral outliers, the number of chiral centers analysed, the number of these observed in the model and the number defined in the Chemical Component Dictionary. Similar counts are reported in the Torsion and Rings columns. '-' means no outliers of that kind were identified.

Mol	Type	Chain	Res	Link	Chirals	Torsions	Rings
2	GOL	B	502	-	-	2/4/4/4	-
3	94Q	B	503	4	-	18/57/88/88	0/4/4/4
2	GOL	A	502	-	-	2/4/4/4	-
3	94Q	A	503	4	-	19/57/88/88	0/4/4/4
2	GOL	A	501	-	-	1/4/4/4	-
2	GOL	B	501	-	-	4/4/4/4	-

All (10) bond length outliers are listed below:

Mol	Chain	Res	Type	Atoms	Z	Observed(Å)	Ideal(Å)
3	B	503	94Q	C50-C49	-3.54	1.31	1.51
3	B	503	94Q	C53-C52	-3.49	1.32	1.51
3	A	503	94Q	C53-C52	-3.46	1.32	1.51
3	A	503	94Q	C50-C49	-3.36	1.32	1.51
3	A	503	94Q	P29-O31	3.18	1.60	1.50
3	B	503	94Q	P29-O31	2.87	1.59	1.50
3	B	503	94Q	P09-O33	2.67	1.60	1.50
3	A	503	94Q	P09-O33	2.35	1.59	1.50
3	A	503	94Q	C59-C60	-2.34	1.49	1.53
3	A	503	94Q	C44-N43	2.34	1.51	1.46

All (23) bond angle outliers are listed below:

Mol	Chain	Res	Type	Atoms	Z	Observed(^{o})	Ideal(^{o})
3	A	503	94Q	C41-C40-N39	-5.42	100.95	111.90
3	A	503	94Q	C37-C03-C05	4.81	116.07	108.23
3	B	503	94Q	P09-O08-P07	-4.80	116.37	132.83
3	A	503	94Q	C40-N39-C38	4.73	131.02	122.59
3	A	503	94Q	P09-O08-P07	-4.58	117.12	132.83
3	A	503	94Q	C02-C38-N39	-4.36	107.90	116.58
3	A	503	94Q	C04-C03-C05	-4.18	101.41	108.23
3	A	503	94Q	C04-C03-C02	4.10	115.92	108.82
3	B	503	94Q	C04-C03-C02	3.61	115.08	108.82
3	B	503	94Q	C50-C49-C48	3.60	126.13	113.19
3	A	503	94Q	C50-C49-C48	3.38	125.33	113.19
3	B	503	94Q	C37-C03-C02	-3.12	103.42	108.82
3	A	503	94Q	C48-C47-S46	2.84	116.76	113.46
3	A	503	94Q	C41-C42-N43	2.81	121.15	116.42
3	B	503	94Q	C41-C40-N39	-2.44	106.97	111.90
3	A	503	94Q	O16-C15-C14	-2.43	103.38	106.93
3	A	503	94Q	C40-C41-C42	-2.32	108.49	112.36
3	A	503	94Q	C14-C13-C12	-2.23	99.26	103.22
3	B	503	94Q	O16-C15-C14	-2.13	103.81	106.93
3	A	503	94Q	C37-C03-C02	-2.09	105.19	108.82
3	A	503	94Q	C54-C53-C52	2.09	125.01	114.42
3	A	503	94Q	O63-C47-C48	-2.08	121.54	123.99
3	B	503	94Q	C54-C53-C52	2.07	124.94	114.42

There are no chirality outliers.

All (46) torsion outliers are listed below:

Mol	Chain	Res	Type	Atoms
3	B	503	94Q	O01-C02-C03-C04
3	B	503	94Q	O01-C02-C38-O65
3	B	503	94Q	C11-O10-P09-O08
3	B	503	94Q	C13-O28-P29-O32
2	A	502	GOL	O1-C1-C2-C3
3	A	503	94Q	C38-C02-C03-C04
3	A	503	94Q	C38-C02-C03-C05
3	A	503	94Q	C38-C02-C03-C37
3	A	503	94Q	O01-C02-C03-C04
3	A	503	94Q	O01-C02-C03-C05
3	A	503	94Q	O01-C02-C03-C37
3	A	503	94Q	N43-C44-C45-S46
3	A	503	94Q	C53-C54-C55-C56
3	A	503	94Q	C05-O06-P07-O08
2	B	501	GOL	O1-C1-C2-O2
2	B	501	GOL	O1-C1-C2-C3
2	A	502	GOL	O1-C1-C2-O2
3	B	503	94Q	C50-C51-C52-C53
2	B	502	GOL	C1-C2-C3-O3
2	A	501	GOL	O1-C1-C2-C3
2	B	501	GOL	C1-C2-C3-O3
3	B	503	94Q	C53-C54-C55-C56
2	B	501	GOL	O2-C2-C3-O3
3	B	503	94Q	O10-C11-C12-C13
3	B	503	94Q	N39-C40-C41-C42
3	A	503	94Q	C04-C03-C05-O06
3	B	503	94Q	O01-C02-C03-C37
3	B	503	94Q	P09-O08-P07-O35
3	B	503	94Q	C51-C52-C53-C54
3	A	503	94Q	O63-C47-S46-C45
3	B	503	94Q	C13-O28-P29-O31
3	A	503	94Q	C37-C03-C05-O06
3	A	503	94Q	P07-O08-P09-O33
3	B	503	94Q	C11-O10-P09-O34
3	A	503	94Q	C05-O06-P07-O35
3	B	503	94Q	O01-C02-C03-C05
3	A	503	94Q	O01-C02-C38-O65
3	B	503	94Q	C48-C49-C50-C51
3	B	503	94Q	C52-C53-C54-C55
2	B	502	GOL	O2-C2-C3-O3
3	B	503	94Q	O01-C02-C38-N39
3	A	503	94Q	O01-C02-C38-N39
3	A	503	94Q	C48-C47-S46-C45

Continued on next page...

Continued from previous page...

Mol	Chain	Res	Type	Atoms
3	A	503	94Q	C50-C51-C52-C53
3	A	503	94Q	C11-O10-P09-O08
3	B	503	94Q	C11-O10-P09-O33

There are no ring outliers.

No monomer is involved in short contacts.

The following is a two-dimensional graphical depiction of Mogul quality analysis of bond lengths, bond angles, torsion angles, and ring geometry for all instances of the Ligand of Interest. In addition, ligands with molecular weight > 250 and outliers as shown on the validation Tables will also be included. For torsion angles, if less then 5% of the Mogul distribution of torsion angles is within 10 degrees of the torsion angle in question, then that torsion angle is considered an outlier. Any bond that is central to one or more torsion angles identified as an outlier by Mogul will be highlighted in the graph. For rings, the root-mean-square deviation (RMSD) between the ring in question and similar rings identified by Mogul is calculated over all ring torsion angles. If the average RMSD is greater than 60 degrees and the minimal RMSD between the ring in question and any Mogul-identified rings is also greater than 60 degrees, then that ring is considered an outlier. The outliers are highlighted in purple. The color gray indicates Mogul did not find sufficient equivalents in the CSD to analyse the geometry.

Ligand 94Q B 503

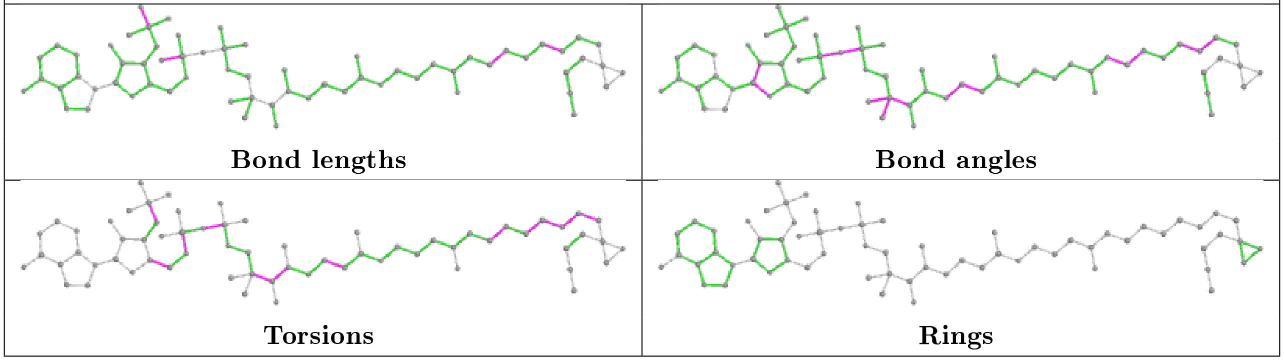

Ligand 94Q A 503

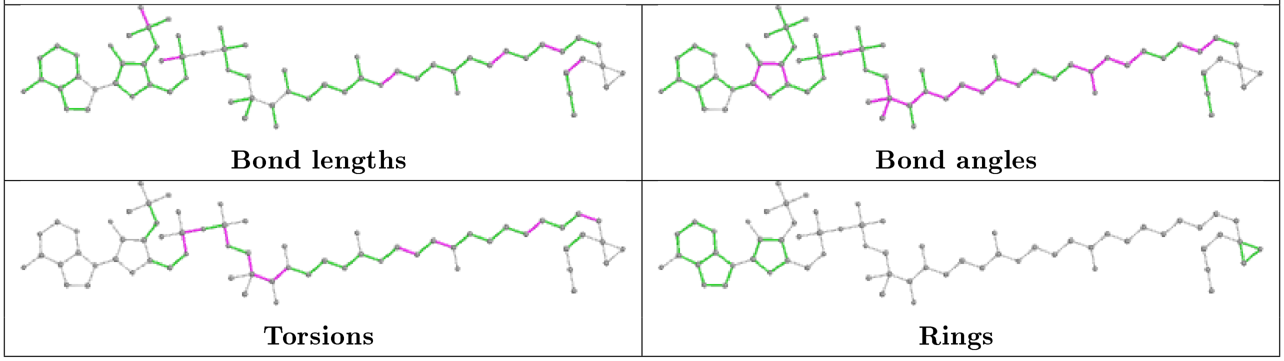

5.7 Other polymers (i)

There are no such residues in this entry.

5.8 Polymer linkage issues (i)

The following chains have linkage breaks:

Mol	Chain	Number of breaks
1	A	1

All chain breaks are listed below:

Model	Chain	Residue-1	Atom-1	Residue-2	Atom-2	Distance (Å)
1	A	147:GLN	C	148:GLU	N	1.17

6 Fit of model and data (i)

6.1 Protein, DNA and RNA chains (i)

In the following table, the column labelled '#RSRZ> 2' contains the number (and percentage) of RSRZ outliers, followed by percent RSRZ outliers for the chain as percentile scores relative to all X-ray entries and entries of similar resolution. The OWAB column contains the minimum, median, 95th percentile and maximum values of the occupancy-weighted average B-factor per residue. The column labelled 'Q< 0.9' lists the number of (and percentage) of residues with an average occupancy less than 0.9.

Mol	Chain	Analysed	<RSRZ>	#RSRZ>2			OWAB(Å^2)	Q<0.9
1	A	355/391 (90%)	-0.25	3 (0%)	86	86	19, 30, 62, 84	0
1	B	375/391 (95%)	-0.14	5 (1%)	77	78	20, 34, 60, 84	0
All	All	730/782 (93%)	-0.19	8 (1%)	80	81	19, 32, 61, 84	0

All (8) RSRZ outliers are listed below:

Mol	Chain	Res	Type	RSRZ
1	A	327	TYR	3.9
1	B	323	THR	3.7
1	A	181	VAL	3.1
1	B	465	LEU	2.5
1	B	324	MET	2.4
1	B	327	TYR	2.4
1	A	320	MET	2.2
1	B	131	VAL	2.1

6.2 Non-standard residues in protein, DNA, RNA chains (i)

There are no non-standard protein/DNA/RNA residues in this entry.

6.3 Carbohydrates (i)

There are no carbohydrates in this entry.

6.4 Ligands (i)

In the following table, the Atoms column lists the number of modelled atoms in the group and the number defined in the chemical component dictionary. The B-factors column lists the minimum,

median, 95th percentile and maximum values of B factors of atoms in the group. The column labelled 'Q< 0.9' lists the number of atoms with occupancy less than 0.9.

Mol	Type	Chain	Res	Atoms	RSCC	RSR	B-factors($Å^2$)	Q<0.9
2	GOL	A	502	6/6	0.89	0.20	29,36,42,46	0
4	MG	B	504	1/1	0.93	0.18	43,43,43,43	0
2	GOL	B	501	6/6	0.94	0.19	29,32,36,39	0
2	GOL	B	502	6/6	0.95	0.17	29,33,36,41	0
3	94Q	A	503	65/65	0.96	0.14	20,32,37,42	0
3	94Q	B	503	65/65	0.96	0.13	24,36,44,47	0
2	GOL	A	501	6/6	0.97	0.13	24,27,30,30	0
4	MG	A	504	1/1	0.98	0.21	38,38,38,38	0

The following is a graphical depiction of the model fit to experimental electron density of all instances of the Ligand of Interest. In addition, ligands with molecular weight > 250 and outliers as shown on the geometry validation Tables will also be included. Each fit is shown from different orientation to approximate a three-dimensional view.

Electron density around 94Q A 503:

2mF$_o$-DF$_c$ (at 0.7 rmsd) in gray
mF$_o$-DF$_c$ (at 3 rmsd) in purple (negative)
and green (positive)

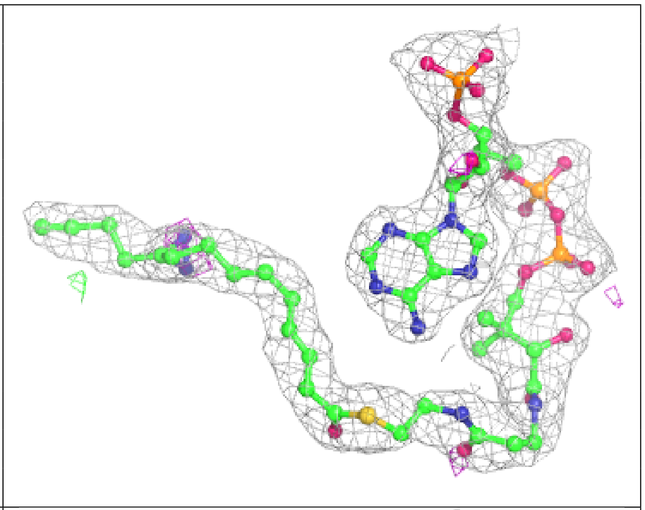

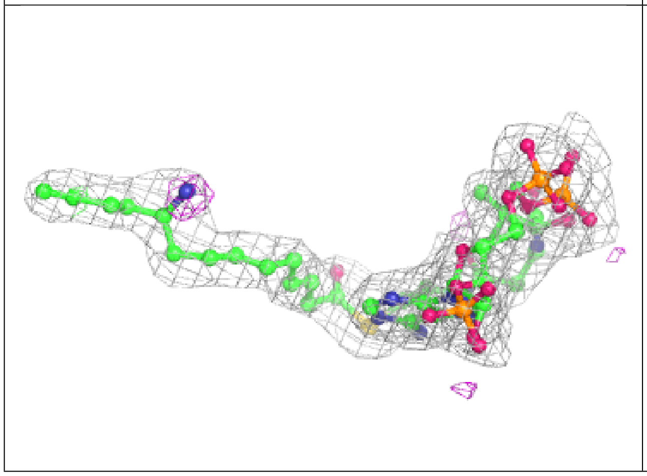

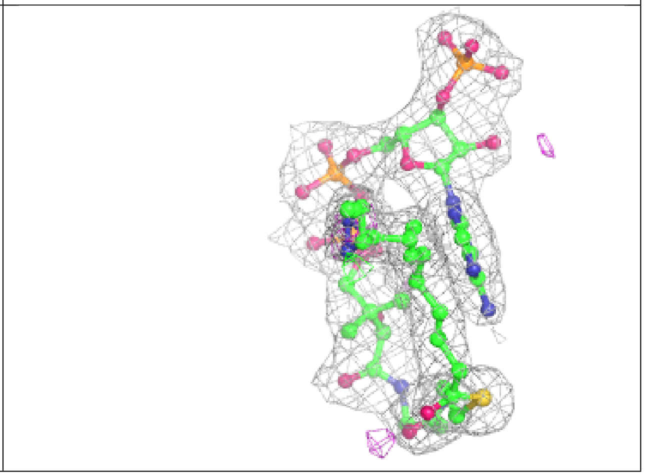

Electron density around 94Q B 503:

2mF$_o$-DF$_c$ (at 0.7 rmsd) in gray
mF$_o$-DF$_c$ (at 3 rmsd) in purple (negative)
and green (positive)

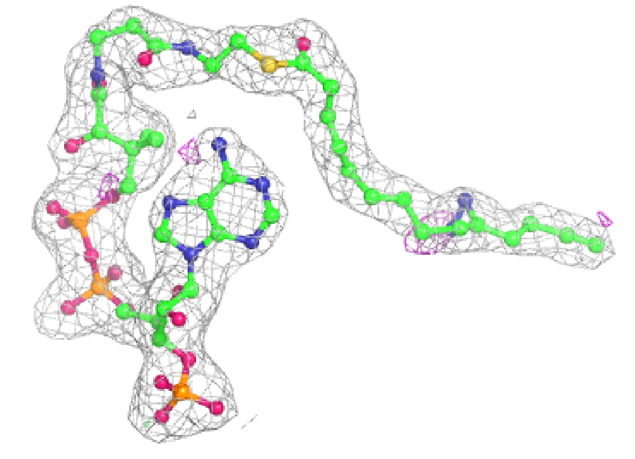

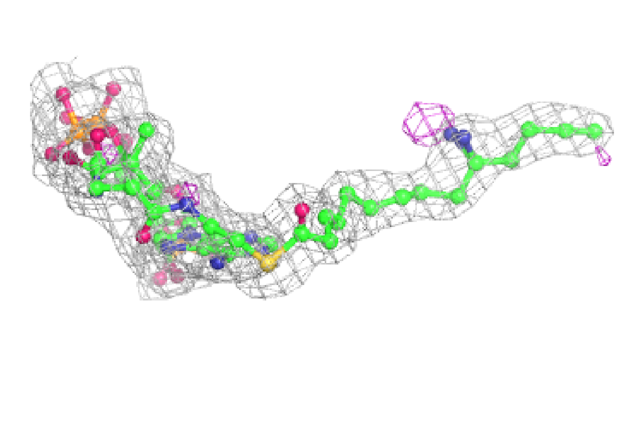

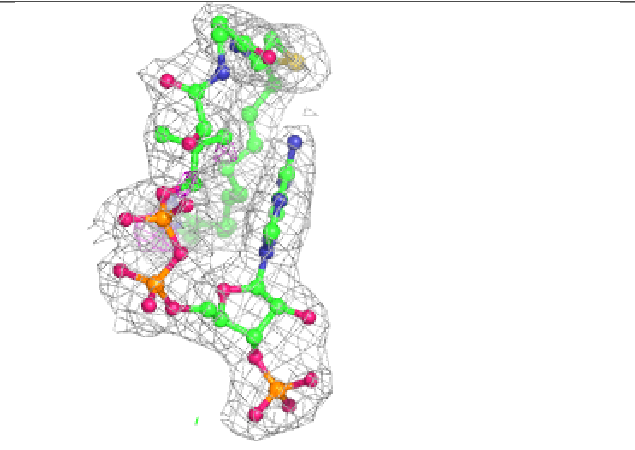

6.5 Other polymers (i)

There are no such residues in this entry.
